# Linking Stochastic Fluctuations in Chromatin Structure and Gene Expression

**DOI:** 10.1371/journal.pbio.1001621

**Published:** 2013-08-06

**Authors:** Christopher R. Brown, Changhui Mao, Elena Falkovskaia, Melissa S. Jurica, Hinrich Boeger

**Affiliations:** Department of Molecular, Cell and Developmental Biology, University of California, Santa Cruz, California, United States of America; University of Massachusetts Medical School, United States of America

## Abstract

The number of mRNA and protein molecules expressed from a single gene molecule fluctuates over time. These fluctuations have been attributed, in part, to the random transitioning of promoters between transcriptionally active and inactive states, causing transcription to occur in bursts. However, the molecular basis of transcriptional bursting remains poorly understood. By electron microscopy of single *PHO5* gene molecules from yeast, we show that the “activated” promoter assumes alternative nucleosome configurations at steady state, including the maximally repressive, fully nucleosomal, and the maximally non-repressive, nucleosome-free, configuration. We demonstrate that the observed probabilities of promoter nucleosome configurations are obtained from a simple, intrinsically stochastic process of nucleosome assembly, disassembly, and position-specific sliding; and we show that gene expression and promoter nucleosome configuration can be mechanistically coupled, relating promoter nucleosome dynamics and gene expression fluctuations. Together, our findings suggest a structural basis for transcriptional bursting, and offer new insights into the mechanism of transcriptional regulation and the kinetics of promoter nucleosome transitions.

## Abstract 2

Electron microscopy of single gene molecules and mathematical modeling shows that a promoter stochastically transitions between transcriptionally favorable and unfavorable nucleosome configurations, providing a mechanism for transcriptional bursting.

## Author Summary

In eukaryotes, such as plants, fungi, and animals, the DNA is wrapped around basic protein cores called nucleosomes at more or less regular intervals. This wrapping discourages transcription, the first step in gene expression. By isolating *PHO5* gene molecules from yeast cells and analyzing their structure by electron microscopy, we provide evidence that the “nucleosomes” completely unwrap and then re-wrap in an intrinsically stochastic manner. Only nucleosomes that wrap the regulatory sequences of the gene (promoter) were observed to unspool; no such unspooling was found across the body of the gene. Random unwrapping and re-wrapping generates an ensemble of alternative promoter nucleosome configurations, some conducive to transcription, others not. Mounting evidence suggests that transcription occurs in bursts, where transcripts are released in close succession, interrupted by intervals of transcriptional inactivity; this may lead to significant stochastic fluctuations in gene expression. Although the mechanism of this behavior is not understood, our findings now provide a structural basis for it, suggesting that spooling and unspooling of promoter DNA from the nucleosomes determines the fundamental frequency of transcriptional bursting.

## Introduction

The number of gene product molecules fluctuates over time and between cells [1]. The magnitude of such fluctuations (“expression noise”) is generally expressed in terms of variance over the mean of gene expression (“Fano factor”), or variance over mean squared (“coefficient of variation” [*CV^2^*]). Gene expression may be viewed as a sequence of molecular transitions; two noise components are then distinguishable: “intrinsic noise,” which derives from the random choice between alternative transitions and the statistical distribution of dwell times between transitions, and “extrinsic noise,” which arises from fluctuations in the cellular concentrations of the biochemical factors that promote the transitions [2].

**Figure 1:**
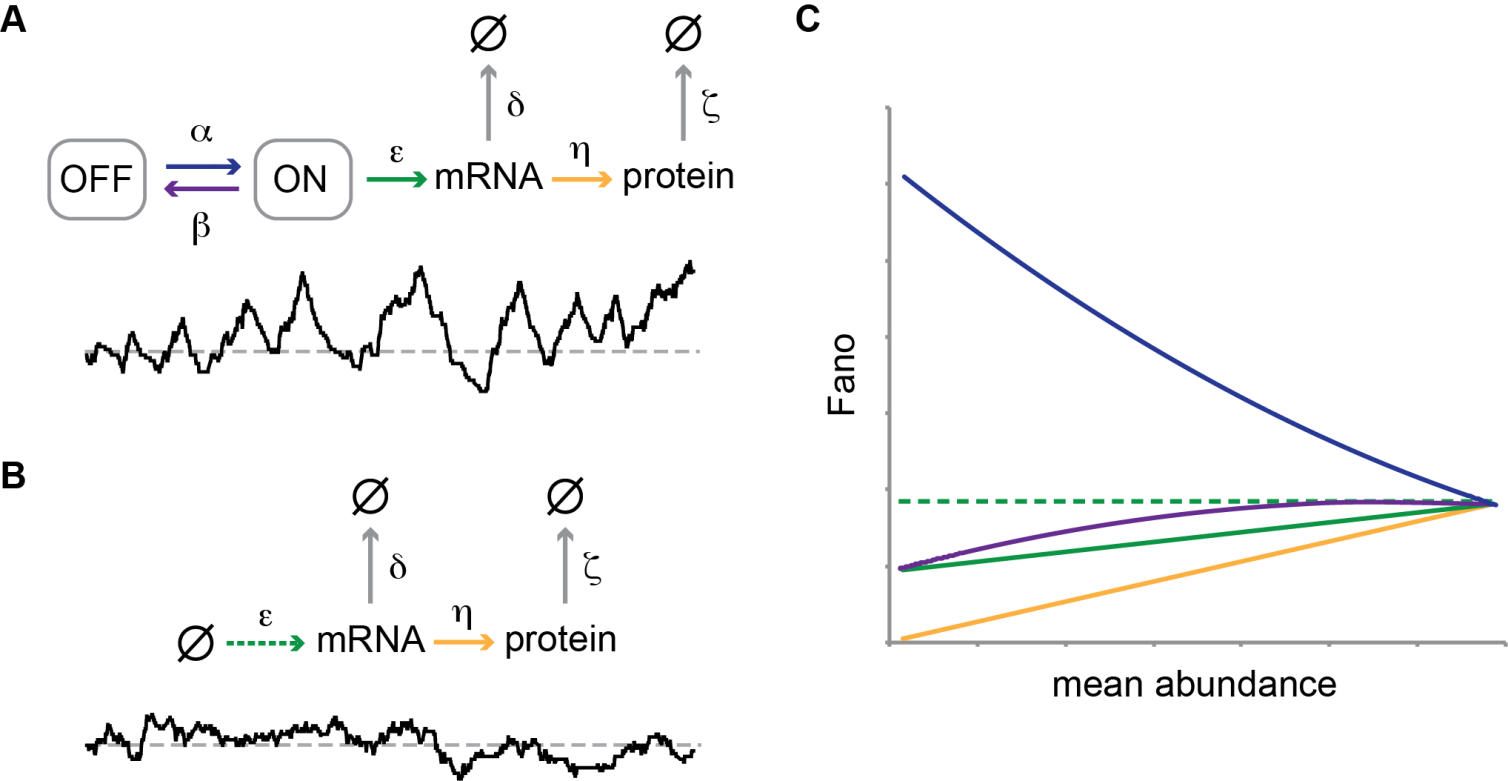
Different modes of gene regulation predict distinct expression noise profiles. (A) The “two-state model” of stochastic gene expression. The model simplifies promoter state dynamics into the stochastic transitioning between two states, ON (transcriptionally active), and OFF (inactive). Transitions→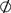 indicate degradation of the gene product. Greek letters refer to transition probabilities per unit time and molecule (“kinetic parameters”); below, a typical time trace (black curve) for the fluctuation in single cell mRNA molecule number about the steady state mean (dashed gray line). (B) “Deterministic model” of a transcriptionally active gene. The black curve beneath the model represents a typical time trace of mRNA fluctuation about the same mean (dashed gray line) as in (A). (C) Steady-state Fano factor values (Fano) were calculated as a function of a single kinetic parameter (the “regulatory parameter”), with all other kinetic parameters held constant. The coloring of the resulting noise profiles refers to the identity of the kinetic parameter that was allowed to float to vary the mean abundance of protein molecules (mean abundance). Thus, blue refers to the bursting frequency α, see (A), etc. The dashed green line indicates the expected Fano profile for the modulation of ε for the deterministic model B. Noise profiles were determined by analytical calculations as described in Materials and Methods.

Stochastic models have been proposed to account for the intrinsic noise of gene expression. An essential component of such models is the assumption that genes randomly transition between states that are either transcriptionally active (“ON state”), or inactive (“OFF state”) [3]. In the ON state a “burst” of transcripts is released, whose magnitude both depends on the rate of transcription and the average life-time of the ON state (Figure 1A). The notion of transcriptional bursting contrasts with the conventional (“deterministic”) model of a transcriptionally active gene, which assumes continual competence for transcription, and where regulation of mRNA synthesis is limited to changes in the rate of transcription (Figure 1B). In the stochastic model (Figure 1A), transcriptional activators may stimulate transcription by either modulating the frequency of transcriptional bursting, burst size, or both. Which of these possible mechanisms is employed may be tested by measurements of intrinsic noise as a function of the average gene product abundance, for model calculations indicate that the intrinsic noise changes in characteristic ways depending on the identity of the steps that are tuned to alter expression (Figure 1C) [4]. However, the molecular basis for transcriptional bursting is not understood, and alternative mechanisms, such as random partitioning of mRNAs at cell division, have been proposed to account for the observed variability in gene product abundance [5].

The wrapping of DNA into nucleosomes limits access of activators and general transcription factors to promoter DNA and impedes the transcribing RNA polymerase [6]–[9]. Consistently, *in vivo* studies support the notion that nucleosomes are general repressors of transcription [10],[11]. The inducible *PHO5* promoter of yeast has served as a classical paradigm for studies of the relationship between promoter chromatin structure and transcription [12]. Structural studies of the *PHO5* promoter pointed at the possibility of alternative nucleosome configurations for the induced *PHO5* promoter, however, without directly demonstrating their existence [13]–[15]. These and other observations have given rise to the notion that fluctuations in promoter chromatin structure might underlie transcriptional bursting [4],[13],[16]. Critical testing of this theory calls for the analysis of promoter chromatin structure at the level of single gene molecules, rather than molecule ensemble averages after endonucleolytic cleavage, the conventional experimental approach.

Here, we report the analysis of the promoter nucleosome configuration of single *PHO5* gene molecules by electron microscopy (EM). Our data demonstrate the existence of alternative promoter nucleosome configurations at steady state *PHO5* expression, including the maximally repressive, fully nucleosomal, and maximally non-repressive, nucleosome-free configuration. We show that the observed configurational probability distribution of *PHO5* promoter nucleosomes is obtained by a simple stochastic process of nucleosome assembly, disassembly, and position-specific sliding. Our analysis thus provides a molecular basis for transcriptional bursting; and we confirm that *PHO5* expression indeed bears the signature of such bursting, with bursting frequency, rather than burst size, as the parameter that responds to transcriptional activators. We demonstrate the possibility of an integrated model of promoter chromatin dynamics and gene expression that quantitatively accounts for measurements of gene expression noise and EM data. The model allows us to predict the life times of microscopically observable promoter nucleosome configurations under repressing and activating conditions, and identifies specific promoter nucleosome transitions as essential for activated transcription.

## Results

### Single Molecule Analysis of *PHO5* Chromatin Structure

**Figure 2:**
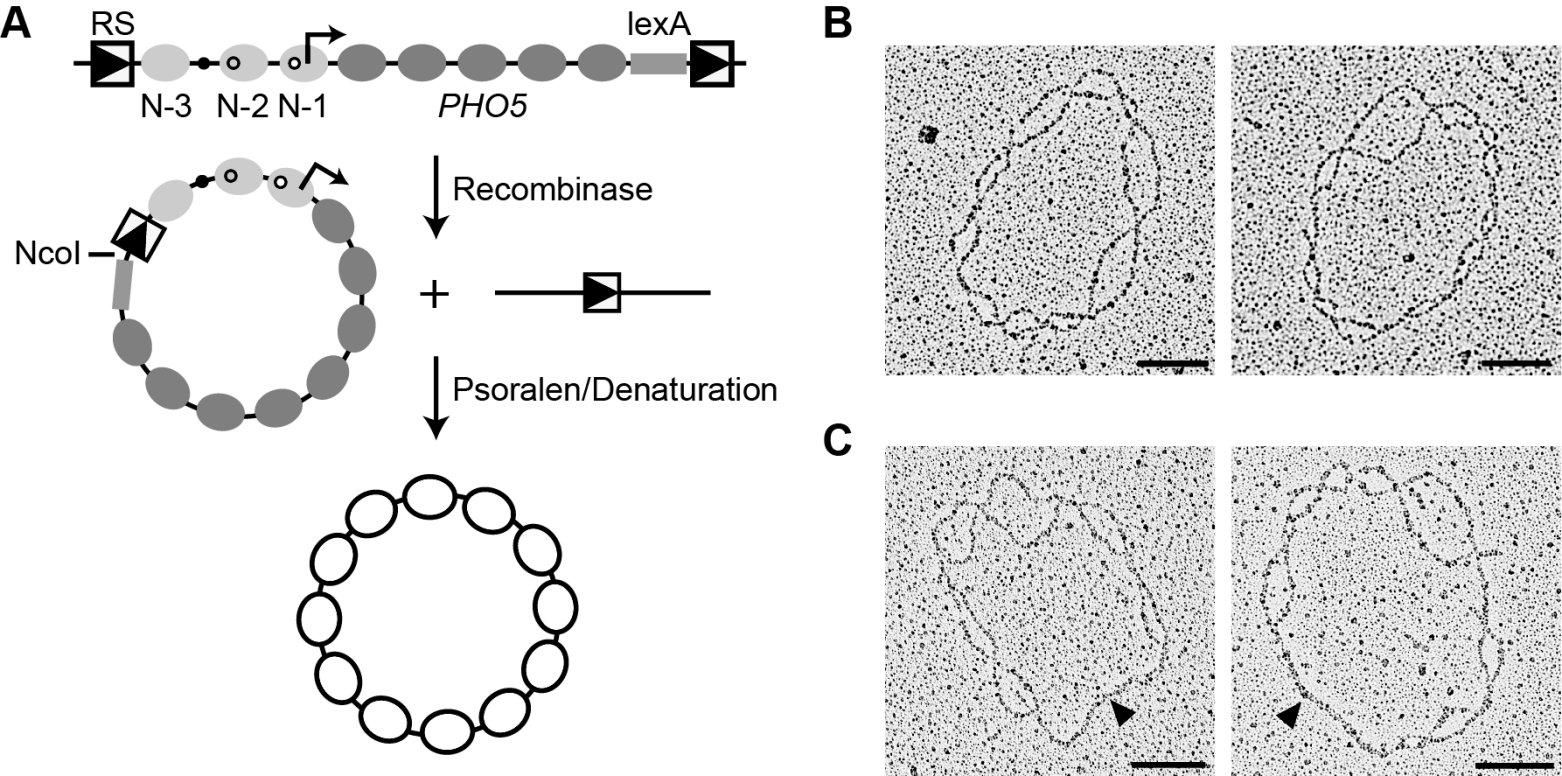
EM analysis of single gene molecules. (A) *PHO5* gene chromatin rings were formed by site-specific recombination *in vivo* [14]. Isolated chromatin rings were crosslinked with psoralen, denatured, and analyzed by EM. Positions of UASp1, UASp2, and the TATA box are indicated by a black, gray, and white circles, respectively; gray ovals represent nucleosomes; promoter nucleosomes are in light gray; *RS* refers to the recognition sequence for site-specific recombination; and *lexA* refers to a cluster of LexA operators for ring purification. (B) EM images of transcriptionally inactive *PHO5* rings (*pho4*Δ *pho80*Δ). (C) EM images of transcriptionally active *PHO5* rings (*PHO4 pho80*Δ). Black arrowheads indicate nucleosome-free DNA segments long enough to accommodate one or more nucleosomes. Bars denote 100 nm.

To analyze the nucleosome configuration of single *PHO5* gene molecules, we isolated chromatin rings encompassing the promoter nucleosome positions N-3 to N-1 and the open reading frame of the yeast *PHO5* gene (Figure 2A). Chromatin rings were formed *in vivo* by induction of site-specific recombination between recombination sequences (RS) flanking the *PHO5* locus (Figure 2A) [14]. A cluster of lexA operator sequences allowed for ring purification by expression of an adaptor protein that contained LexA fused to a tandem affinity tag [17]. The promoter DNA of purified chromatin rings and isolated nuclei exhibit closely similar sensitivities to restriction endonucleases, suggesting essentially identical chromatin structures [14],[18]; and *PHO5* is fully inducible after ring formation (Figure S3). Transcriptionally “active” *PHO5* molecules were isolated from *pho80*Δ cells in which *PHO5* is expressed constitutively [19].

Purified chromatin rings were incubated with trimethylpsoralen and exposed to ultraviolet (UV) light, resulting in inter-strand crosslinking of the DNA double helix in nucleosomal linkers, but not core particle DNA [20], thus “etching” nucleosome configurations into the DNA. Purified ring DNA was denatured, spread onto ethidium-coated carbon grids, stained with uranyl acetate, and rotary metal shadowed prior to EM. Positions previously occupied by nucleosome core particles thus appear as single-stranded DNA bubbles, connected by double stranded linker DNA that resisted denaturation due to crosslinking [20].

**Figure 3:**
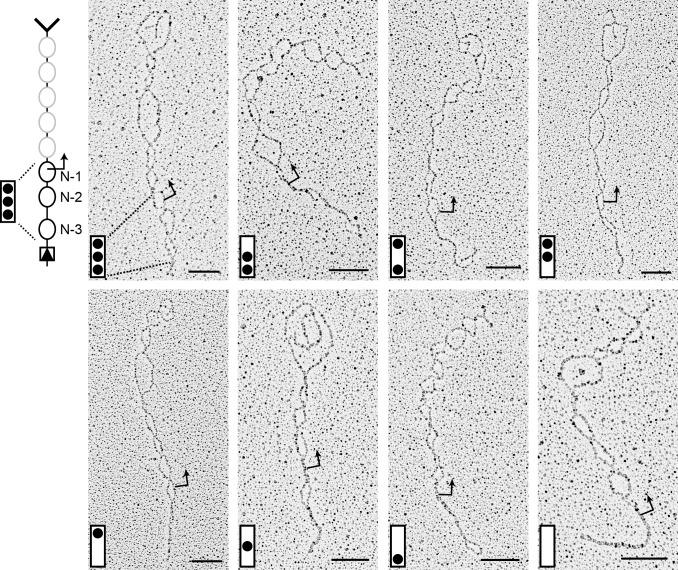
Nucleosome configurations of “activated” promoters. *PHO5* gene molecules are aligned with their 3′ forked end on the top. A bent arrow indicates the position of the transcription start site. The inferred promoter nucleosome configuration is shown in the left lower corner of each image, where the promoter is represented by a box and occupied nucleosome positions by black dots. The top position represents N-1, the middle position N-2, and the bottom position N-3. Nucleosome configurations representing all eight possible combinations of occupied and unoccupied positions N-1 to N-3 were observable. Bars indicate 100 nm. (See also Figure S1).

Single-stranded DNA bubbles were densely spaced on *PHO5* rings isolated from repressed cells, separated by only short segments of crosslinked double stranded DNA (Figure 2B). Larger than expected bubbles suggested fusion of nucleosome bubbles due to failure of crosslinking of the intervening linker DNA, which is rather short in the open reading frame of the gene (Materials and Methods). In contrast, the majority of *PHO5* rings isolated from activated cells exhibited larger contiguous segments of crosslinked DNA (Figure 2C). To determine whether these continuously crosslinked regions coincided with promoter sequences, we linearized rings by NcoI restriction enzyme digestion (Figures 2A and 3). Larger nucleosome-free segments were found only at or close to one end of linearized rings, and not in their interior (Figure 3). At the opposite end, which bears the lexA operator cluster, molecules were forked, suggesting that the binding of LexA adaptor proteins at the cluster prevented its crosslinking. Consistently, forked ends were not observed in control experiments with naked DNA molecules (Figure S1). The fork thus oriented molecules, and enabled us to identify the promoter nucleosome bubbles on all *PHO5* gene molecules.

**Figure 4:**
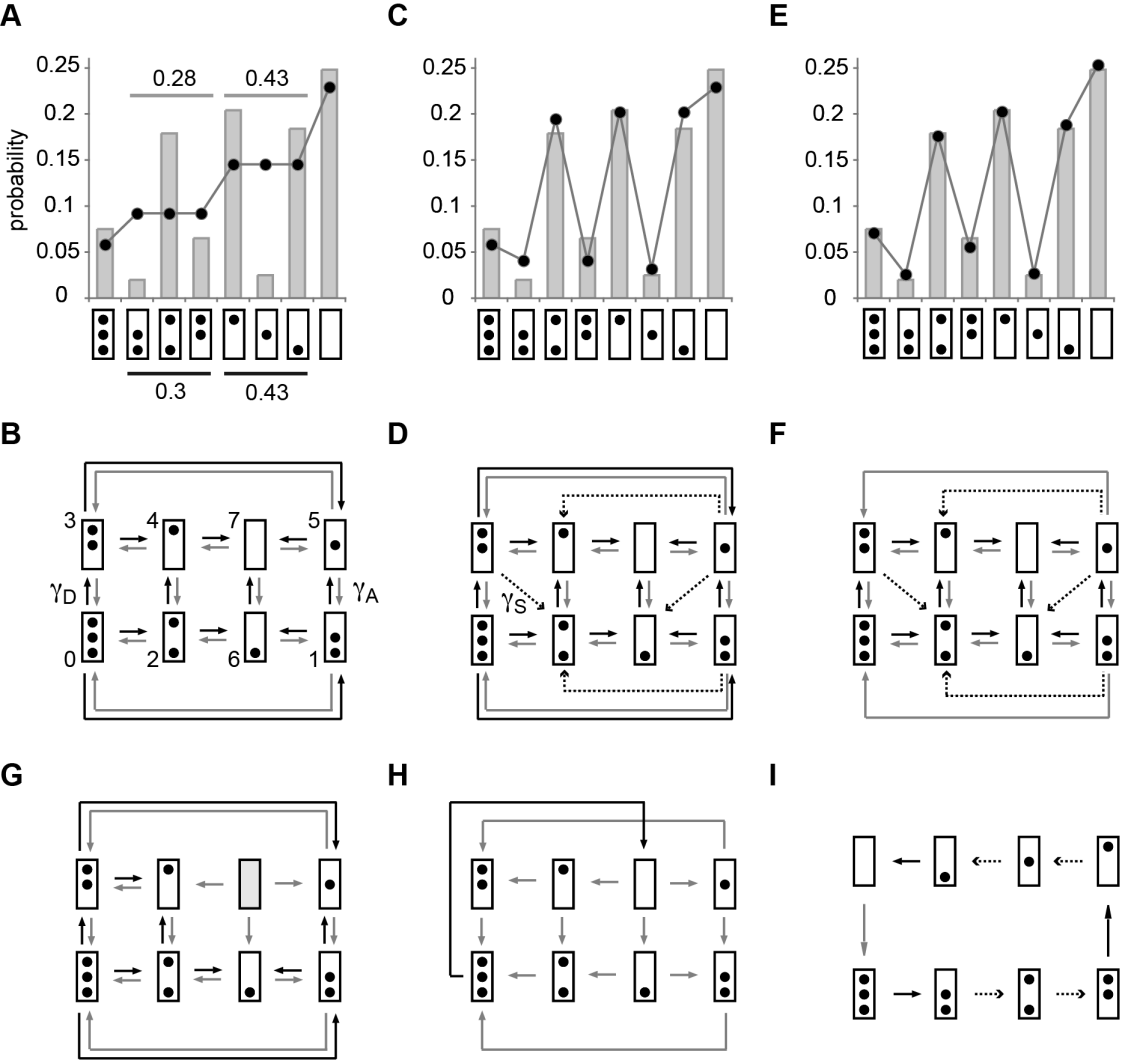
Probabilities of promoter nucleosome configurations. Bars indicate the relative frequencies of promoter nucleosome configurations (“probability”) for EM datasets (Table S1). Model predictions are indicated by black dots, connected by gray edges to aid the visual comparison with EM data. Promoter nucleosome configurations are represented as in Figure 3. (A) Configurational probability distribution of *PHO5* promoter nucleosomes in activated cells (*PHO4 pho80*Δ). Numbers above and below horizontal lines refer to the sum of probabilities for 2-nucleosome and 1-nucleosome configurations, determined by EM (above light gray line), or model calculation (below dark gray bar). Predictions were based on the transition topology in (B). (The same predictions were obtained for a model with “symmetric sliding,” which allows for all possible sliding transitions.) (B) Transition topology without nucleosome sliding; nucleosome assembly and disassembly transitions are indicated by gray and black arrows, respectively. (C) Same as (A), however with predictions based on the topology in (D). The statistical support of the topology in (D) against its rival hypothesis in (B) given the EM dataset *R* was 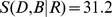; i.e., *R* was 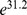-fold more probable given (D) than given (B) (Materials and Methods). (D) Transition topology with unidirectional nucleosome sliding; dashed arrows indicate sliding transitions. (E) Same as (A), with predictions based on the topology in (F); 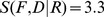 (and hence 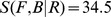). (G) Transition topology for “stable nucleosome retention.” This hypothesis was disproved by *R*, for 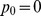, but 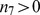; thus, its likelihood, given *R*, was 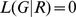. (H) Transition topology for all-or-nothing disassembly: 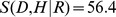. (I) Transition topology for “deterministic cyclical process”; 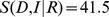. The transition topologies in (G) to (I) were refuted given the strong support for topologies in (D) and (F) against their rival hypotheses. For parameter values see Table S3.

Eight promoter nucleosome configurations were observed, representing all combinations of occupied and unoccupied positions N-1 to N-3 (Figure 3). To assign nucleosome bubble identities and thus determine the relative frequency of each promoter nucleosome configuration, we determined for each linearized ring the positions of upstream activation sequences 1 (UASp1) and 2 (UASp2; position N-2), and the transcription start site (position N-1) by measuring their expected distance from the proximal DNA end. Both upstream activation sequences bear a binding site for the transcriptional activator Pho4, which is essential for *PHO5* expression [21]. The relative frequencies of promoter nucleosome configurations for *PHO5* rings isolated from *PHO4* wild type cells is shown in Figure 4A. Nucleosome occupancies determined by EM were in good agreement with nuclease accessibility measurements on isolated nuclei [14],[15]. For instance, micrococcal nuclease and restriction endonuclease analysis of position N-1 indicated an occupancy of position N-1 between 0.5 and 0.6 for the “active” promoter, closely similar to the occupancy of 0.52 inferred by EM (Figure S2; Table S1).

### Model of Promoter Nucleosome Dynamics

How can the observed configurational probability distributions of the promoter nucleosomes be explained? In the following we shall demonstrate that the observed probability distributions can be explained as the result of an intrinsically stochastic process, i.e., a process where the future configuration and the configuration's life-time can be predicted only probabilistically. Specifically, we assume that the probability of finding the promoter in state *j* at time *t*+*h*, given that the promoter was in configuration *k* at time *t*, equals 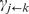 *h* for sufficiently small time intervals *h*, where 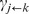, the transition probability per time and molecule, depends only on *j* and *k* (assumption of a time homogeneous Markov process). The steady state probabilities *p*_0_,…, *p*_7_ of nucleosome configurations 0,…, 7 of such a process obey the following matrix equation [13],[15]:
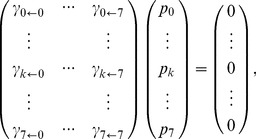
(*i*)with diagonal elements 
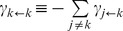
. We refer to the 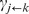
's as the “kinetic parameters” of the process. Experimentally, steady state was achieved by isolating chromatin rings from *pho80*Δ cells (see above). The task at hand is to find 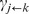
's that are consistent with equation (*i*), or more precisely, a matrix 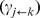 with diagonal elements as above whose kernel is spanned by the observed steady state distribution (*p*_0_,…, *p*_7_), or a vector very similar to it.

Transitions between nucleosome configurations may be due to assembly, disassembly, or sliding of a nucleosome. Accordingly, we may distinguish three different kinds of transitions. We call the stochastic process “simple” if transitions of the same kind have the same kinetic parameter value, 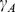 for assembly, 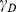 for disassembly, and 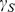 for sliding transitions; the term “transition topology” refers to the set of all possible transitions.

The assumption of a simple process reduces the task of finding the desired matrix 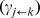 to drawing the “correct” transition topology. A transition topology that is limited to transitions between configurations that differ by one nucleosome, and that has therefore only two kinds of transitions—nucleosome assembly and disassembly—may serve as a starting point (Figure 4B).

This topology, with 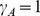 on some appropriate time scale and 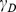 determined by maximum likelihood analysis (Materials and Methods), accounted well for the observed probabilities of nucleosome number, but not the probabilities of individual configurations (Figure 4A). The discrepancy between prediction and experimental observation could thus be attributed to a redistribution of probability mass between nucleosome configurations with equal nucleosome number, and hence to the sliding of nucleosomes between promoter positions. With 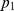 and 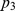 similar, but lower than 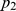; and 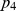 and 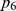 similar, but higher than 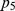 (Figure 4A), the observed distribution suggested sliding of nucleosomes out of position N-2 (Figure 4D; “unidirectional sliding topology”). Indeed, observed probabilities and probabilities calculated on the basis of the transition topology of Figure 4D were closely similar (Figure 4C), with 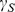 determined by maximum likelihood analysis. Remarkably, the assumption that disassembly of nucleosomes in position N-1 requires a nucleosome-free position N-2 (Figure 4F), suggested by nucleosome accessibility analysis over the time course of *PHO5* induction [22], led to a virtually perfect agreement between measurements and predictions (Figure 4E). In contrast, other conceivable topologies, for instance for “stable nucleosome retention” (Figure 4G) [13], “all-or-none removal” of promoter nucleosomes (Figure 4H), and “cyclical deterministic” processes, such as the topology of Figure 4I, were refuted by our data.

**Figure 5:**
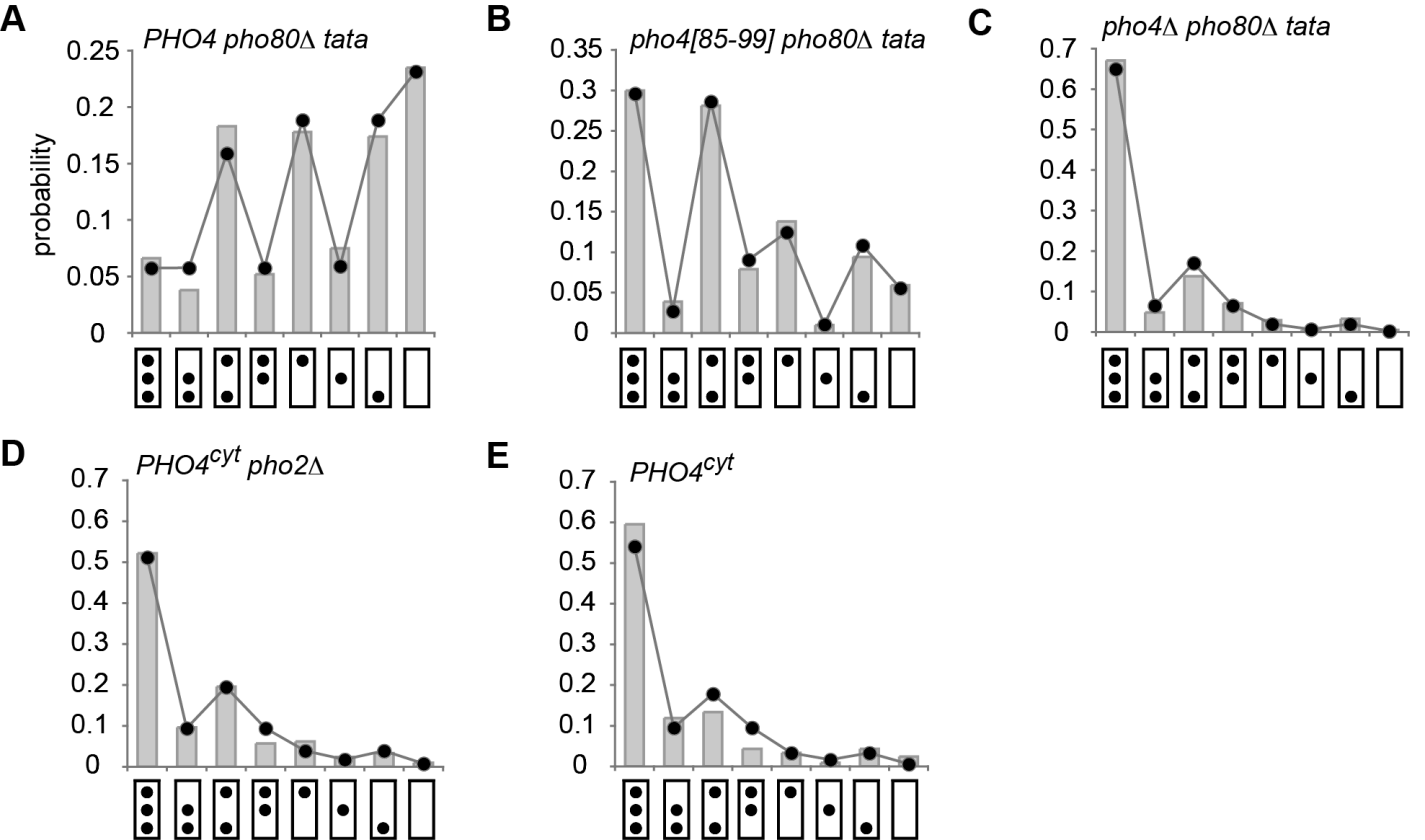
Configurational probability distributions in activator and promoter mutants. (A) Distribution for molecules isolated from *PHO4 pho80*Δ cells with a mutated *PHO5* TATA box (*tata*). Theoretical predictions and experimental results (*R*) are indicated by a chain of dots and histogram bars, respectively, as in Figure 4. Predictions were based on the topology of Figure 4D. The statistical support against its rival of Figure 4F was 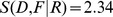. (B) Distribution for molecules isolated from *pho4[85-99] pho80*Δ cells with a mutated *PHO5* TATA box. *PHO5* expression in *pho4[85-99] pho80*Δ cells with wild type *PHO5* TATA is 0.14 relative to *PHO4* wild type (set to 1) [15]. Predictions were based on the topology of Figure 4F, rather than 4D; 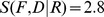. (C) Distribution for molecules isolated from *pho4*Δ *pho80*Δ cells with a mutated *PHO5* TATA box. Expression of *PHO5* in *pho4*Δ *pho80*Δ cells relative to *PHO4* wild type is ∼0.005 (see below and [15]). Predictions were based on the topology of Figure 4D, rather than 4F; 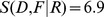. (D) Distribution for molecules isolated from *PHO4 pho2*Δ cells grown in high phosphate (*PHO4^cyt^*); Pho4 is in the cytoplasm, rather than the nucleus, and *PHO5* is repressed, therefore. Predictions were based on the topology of Figure 4D, rather than 4F; 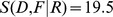. (E) Distribution for molecules isolated from *PHO4* cells grown in high phosphate (*PHO4^cyt^*). Predictions were based on the topology of Figure 4D, rather than 4F; 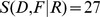. For parameter values see Table S3.

To further test our topological hypotheses, we analyzed the configurational probability distributions in the *PHO5 tata* box mutant (Figure 5A–5C) and strains that bore mutations in *PHO4* or *PHO2* (Figure 5B–5D). (The latter two genes encode the transcriptional activators of *PHO5*.) All observed distributions were well explained by the unidirectional sliding topologies of Figure 4D and 4F; our data generally supported topology 4D over 4F, except for the *pho4*[85–99] mutant, where topology F enjoyed greater statistical support (see legend to Figure 5B). Thus, all observed configurational probability distributions were well explained by a simple stochastic process of nucleosome removal and reformation with no more than two degrees of freedom. Our findings support the hypothesis that the *PHO5* promoter stochastically transitions between alternative nucleosome configurations at steady state.

Notably, while our data clearly indicated a net loss of nucleosomes from the promoter upon induction of *PHO5* (compare Figures 4A and 5C, for instance), no such loss was observed over the open reading frame that could have been attributed to transcription (Table S1). The structural dynamics of promoter and open reading frame nucleosomes appear to be fundamentally different.

### *PHO5* Expression Is Regulated by Changes in Transcriptional Burst Frequency

Nucleosomes inhibit the binding of transcription factors at the *PHO5* promoter [6]. The nucleosome-free and fully nucleosomal configurations are, therefore, either maximally conducive or inconducive to transcription. Hence, our conclusion that the “active” *PHO5* promoter stochastically transitions between the nucleosome-free and fully nucleosomal configuration implies that *PHO5* transcription occurs in random bursts.

**Figure 6:**
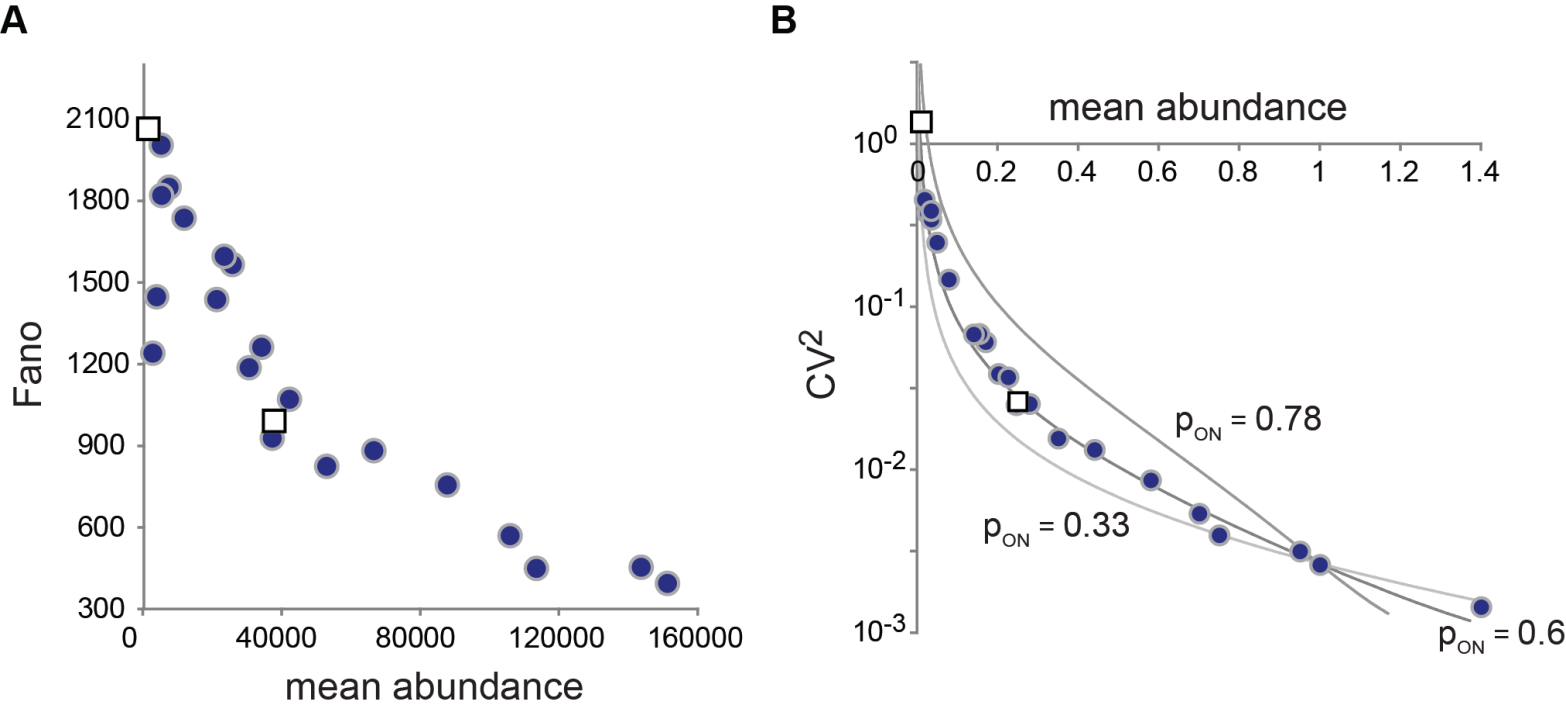
Noise profiles of *PHO5* expression. The results of intrinsic protein noise measurements [15] are indicated by blue dots for strains with mutations in the Pho4 activation domain, and white squares for *pho5* UASp1 and UASp2 mutants. Intrinsic noise was measured [15] using the dual reporter system [1], where cyan fluorescent protein (CFP) and yellow fluorescent protein (YFP) were expressed in diploid cells under control of the *PHO5* promoter. (A) Fano factor profile of *PHO5* protein noise; Fano factor and mean abundance are indicated in molecule numbers, based on the assumption that the average number of protein molecules expressed under repressing conditions is 1,000 per cell [24]. (The exact number is unimportant to our principle conclusions.) (B) *CV* ^2^ profile of *PHO5* protein noise. Mean (protein) abundance is in units of wild type expression. Curves represent predictions based on the two-state model (Figure 1A), with the burst frequency α as the regulatory parameter, and different probabilities of the ON state in wild type (*p*_ON_). For all calculations, 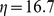 min^−1^, 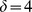 h^−1^, 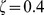 h^−1^ (see Figure 1A, and main text below; like Pho5, CFP and YFP are biochemically stable; the proteins are lost therefore primarily due to dilution by cell division). With 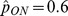, the kinetic parameter for transcription is 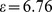 min^−1^. The parameter values were determined as described in the main text.

We previously determined the intrinsic protein noise of *PHO5* expression for 23 mutants that either bore a mutation in the Pho4 activation domain or the upstream activation sequences of the *PHO5* promoter (Figure 6) [15]. In the absence of bursting (Figure 1B), a flat Fano factor profile is expected (Figure 1C). The observed profile, however, deviated significantly from this expectation (Figure 6A), and furthermore suggested that Pho4 regulates *PHO5* expression by modulating the burst frequency of transcription, α, rather than burst size, εβ^−1^ (compare Figures 6A and 1C). We refer to the kinetic parameters that respond to regulatory input, such as α, as “regulatory parameters.”

### Pho4 Accelerates Nucleosome Removal

If burst frequency is the regulatory parameter for *PHO5* expression, then the observed net loss of promoter nucleosomes upon *PHO5* induction is due to accelerated nucleosome removal, and not inhibition of nucleosome assembly; the former changes the frequency of transcriptional bursting, the latter burst size. Comparison of *PHO5* molecules isolated from the TATA box wild type and *tata* box mutant (Figures 4A and 5A), and the *PHO2* wild type and *pho2*Δ mutant (Figure 5D and 5E) allowed for additional testing of this implication. Pho2 binds to several binding sites at position N-2 following nucleosome removal [6] and might thus sterically exclude nucleosomes from position N-2. The same argument applies for the TATA box binding protein (TBP) and the TATA box. However, neither mutation resulted in an increase in nucleosome occupancy at N-2 or N-1, respectively. Our results thus argue against steric exclusion of nucleosomes by transcription factors, and suggest that nucleosome formation does not compete with transcription factor binding. Thus, both noise analysis and EM data corroborate the hypothesis that the net loss of promoter nucleosomes upon transcriptional induction is due to the acceleration of nucleosome removal, and not inhibition of nucleosome assembly, supporting the notion of activator-mediated recruitment of chromatin remodeling factors.

### Which Nucleosome Configurations Are Conducive to Transcription?

Our data show that the majority of “active” *PHO5* molecules exhibited promoter nucleosome configurations between the two extremes of the fully nucleosomal and the nucleosome-free configuration (Figure 4A). The fundamental problem of defining the relationship between promoter chromatin structure and transcription here presents itself again at the single molecule level: which promoter nucleosome configurations, if any, beside the naked configuration might be conducive to transcription? The following considerations show that our data restrict the number of possible answers to this problem (“fundamental problem”).

The transcriptionally active molecules constitute a subset of the transcriptionally conducive ones. It follows that the probability of the ON state, 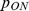, provides a lower bound for the combined probabilities of all nucleosome configurations that are conducive to transcription, 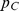, i.e., 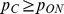.

**Figure 7:**
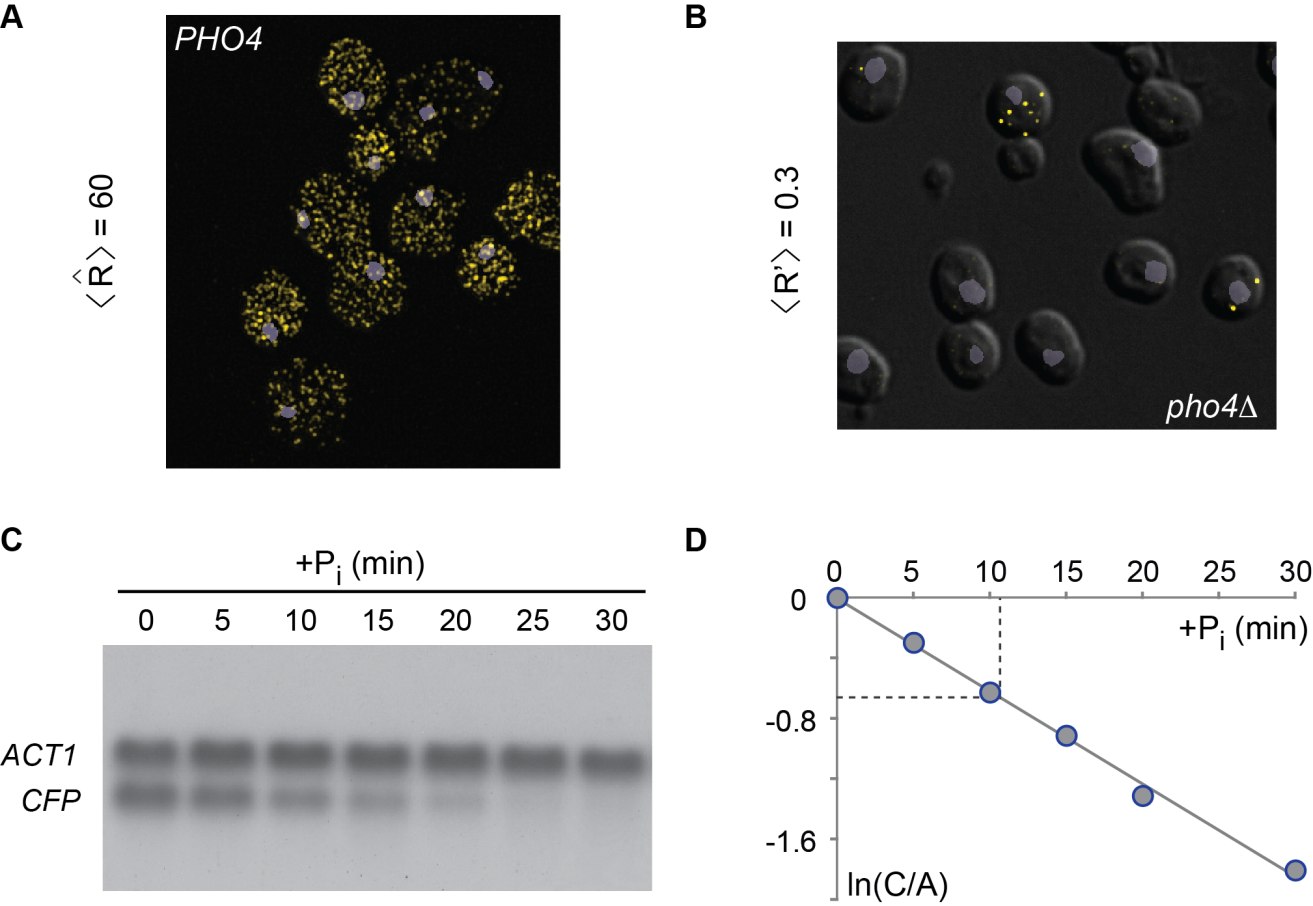
Abundance and half-life of mRNA. (A) FISH of activated cells (*PHO4 pho*80Δ) in which the endogenous *PHO5* promoter drove expression of CFP. For FISH, we used CFP anti-sense DNA oligonucleotides labeled with Alexa 555. Cells contained 60 *CFP*-mRNA molecules, on average. The nucleus was stained with DAPI (blue-gray). (B) FISH of repressed cells (*pho*4Δ *pho*80Δ) revealed an average of ∼0.3 *CFP*-mRNA molecules per cell. (C) The *PHO5* promoter was induced in *PHO80* wild type cells by incubation in phosphate-free medium. RNA samples were taken at different time points following the addition of inorganic phosphate to the medium (+P_i_). RNA was fractionated by agarose gel electrophoresis, blotted, and hybridized with ^32^P-labeled DNA probes against *CFP* and *ACT1* mRNAs. (D) The natural logarithm of the radioactive signal ratio for *CFP* and *ACT1* mRNAs normalized to 1 for 0 minutes of +P_i_ (ln[C/A]) was plotted against the time after addition of phosphate; ln2 is reached at about 10 min, the half life of the transcript, which corresponds to a kinetic parameter for mRNA degradation of 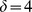 h^−1^. A closely similar value for δ was obtained for the *PHO5* mRNA (data not shown). The steady state abundance of *PHO5* transcripts is therefore expected to be similar to the number of CFP mRNAs per cell.

In the following, we show that 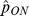, the probability of the ON state, where ∧ refers to the *PHO4* wild type, can be determined from the quantitative relationship between mean protein abundance and the *CV^2^* for the intrinsic protein noise 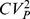, with mean abundance of mRNA molecules in *PHO4* wild type cells 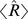, and the kinetic parameters for mRNA degradation δ, protein degradation ζ, and translation η given (Figure 1A). We determined 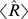 by fluorescence *in situ* hybridization (FISH) (Figure 7A, 7B). The kinetic parameter for mRNA degradation δ, was determined by northern blotting using *PHO80* wild type cells, where the *PHO5* promoter was induced by transferring cells into phosphate-free medium followed by addition of inorganic phosphate to shut down transcription (Figure 7C, 7D). (We assume, here, that the promoter rapidly becomes inactive following addition of phosphate; this assumption is supported by the close fit of the mRNA decay curve to a single exponential function [Figure 7D]; the half life thus determined was identical to the average half life of mRNAs in yeast [23]. Importantly, 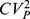 is rather insensitive to changes in δ [Figure S4A]; the inferred value of 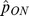 [see below] did not critically depend on δ.)

The parameter for protein degradation, ζ, was calculated from the cell cycle time, as Pho5 activity decays at the rate of cell division (unpublished data). The average number of Pho5 molecules, 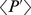, under repressing conditions (indicated by ′) had been determined previously [24]. Together with knowledge of the average number of mRNA molecules, 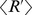, from FISH (Figure 7B), an estimate for the translation parameter η was derived from the steady state condition 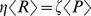.

Like our model of promoter nucleosome dynamics, the stochastic model of gene expression (Figure 1A) is based on the assumption of a time-homogeneous Markov process with transitions between discrete molecular states, defined by the number of mRNA and protein molecules, and the promoter state (ON or OFF) [25]–[27]. Steady state expression noise and mean can be derived analytically for a given set of kinetic parameter values (see Materials and Methods).

For given 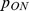, 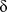 and 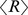, the kinetic parameter for transcription, ε, is provided by the steady state condition 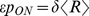. Furthermore, 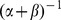, which specifies the time scale of promoter state fluctuations, is determined by 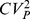. This can be understood intuitively by considering that fast ON/OFF fluctuations, i.e., small 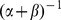, correspond to short dwell times in both ON and OFF states; short dwell times in both states reduce the transcriptional burst size and provide little time for the degradation of gene products during OFF periods, respectively, thus reducing the size of protein molecule number fluctuations about the mean. With 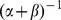 thus determined, and 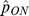 given, α and β can be calculated, using the steady state condition 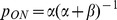.

Arbitrary values for 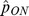 could account for our measurements of 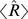 and 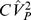 (Figure 6B). However, for 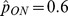, a noise profile was obtained that agreed remarkably well with the observed profile, with the transcriptional burst frequency, α, as regulatory parameter (Figure 6B). The 0.6 value for 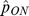 together with our EM data ruled out many conceivable solutions of the ‘fundamental problem’. Indeed, the only set of nucleosome configurations united by a common structural feature with a total probability greater than 0.6, and thus satisfying our previous requirement of 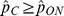, was the set of configurations that lack the nucleosome in position N-2 (configurations 2, 4, 6, and 7; Figure 4B). This result suggests that configurations 2, 4, 6, and 7 are transcriptionally conducive, while configurations 0, 1, 3, and 5 are inconducive.

### Conducive and Active Promoter States

Since the transcriptionally active promoter states are a subset of the states with conducive nucleosome configurations, 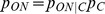, where 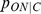 is the probability of the active state, given that the promoter exhibits a transcriptionally conducive nucleosome configuration. If promoter states with a conducive nucleosome configuration were transcriptionally active, i.e., 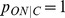 at all times, and thus 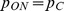, *PHO5* expression would exclusively be controlled by modulation of the kinetic parameters for nucleosome disassembly and sliding; and steady state 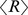 would be a linear function of 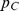: 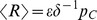. However, while FISH analysis indicated that 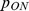 increased ∼200-fold upon *PHO5* induction (Figure 7A, 7B), our EM data showed that 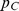 rose by a factor of about 4 only (Figure 4; Table S1)—too little to explain the observed increase in *PHO5* transcription. It follows that 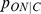 increased by a factor of ∼50 upon *PHO5* induction; this factor indicates the increase in transcription that remains unexplained by promoter chromatin remodeling. We conclude that the Pho4 activator stimulates one or more additional steps of the expression process following nucleosome removal.

### Integrated Model of *PHO5* Expression and Chromatin Dynamics

**Figure 8:**
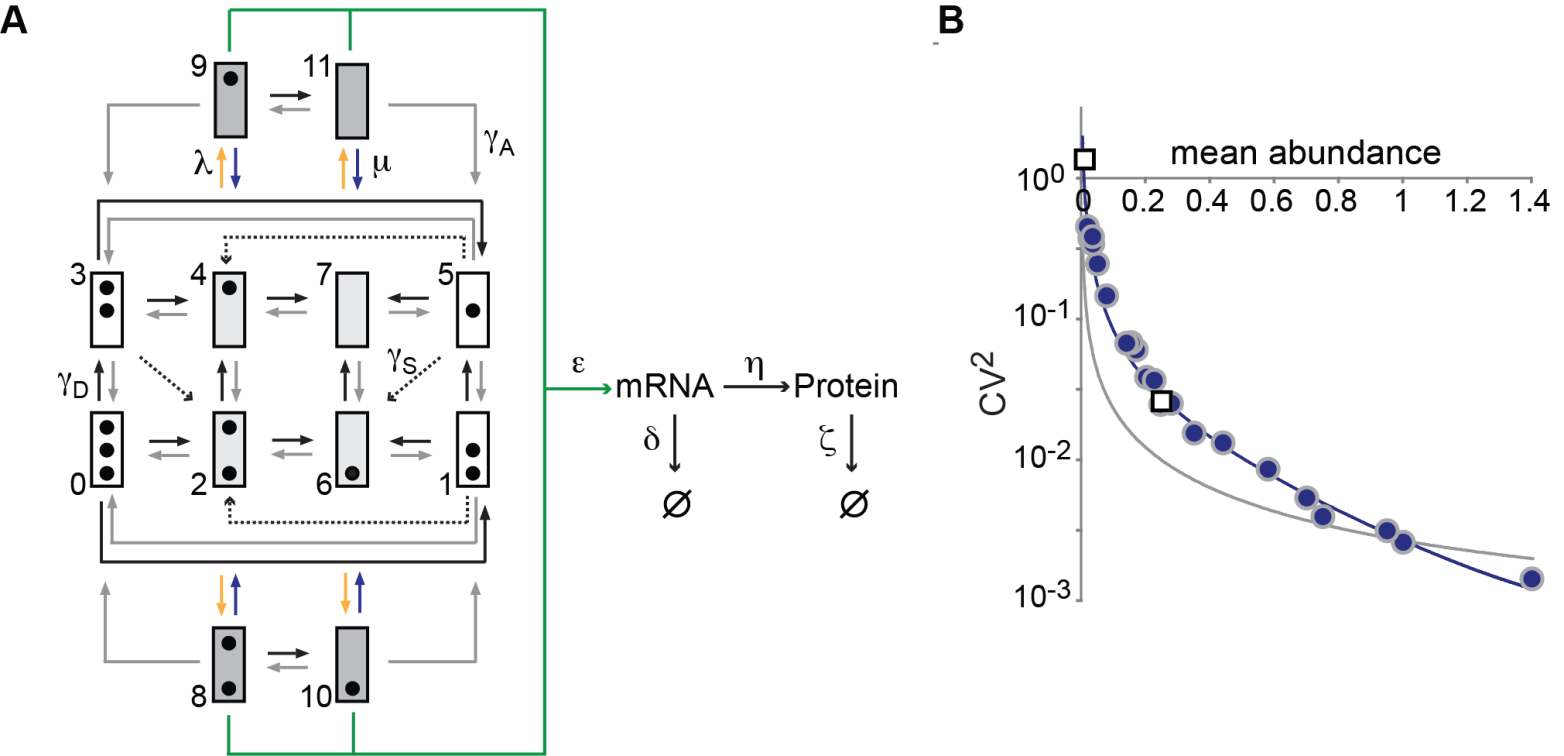
Integrated model of promoter nucleosome dynamics and gene expression. (A) Transition topology of the integrated model. Promoter states are represented by boxes, and black dots indicate the nucleosome configuration of the state, as in Figures 3–5. Promoter states with transcriptionally conducive nucleosome configuration are shaded gray, where light gray promoter states are conducive, and dark gray states are active states; inconducive states are represented by white boxes. Nucleosome assembly and disassembly transitions between states 8 and 9, and 10 and 11 were omitted for graphical clarity. (B) The data are the same as in Figure 6 [15], but the predicted noise profile (blue curve) was calculated (Materials and Methods) using the integrated model with 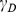, 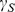, and λ as regulatory parameters, which were allowed to float along the line 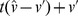, with 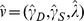, 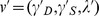, where the hat and prime mark the parameter values for the *PHO4* wild type and *pho4*Δ mutant, respectively, and *t* is a real number ≥0. Noise predictions based on the assumption of 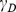, 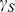, and ε as regulatory parameters, and thus assuming a combination of burst size and burst frequency control, are indicated by the gray curve. Virtually the same result was obtained on the topology of Figure 4F for nucleosome transition (not shown).

A stochastic process based on the transition topology of Figure 8A integrates our findings: Beside the transitions between nucleosome configurations according to the topology of Figure 4D, the model encompasses transitions between transcriptionally active and inactive promoter states. The model thus introduces two additional kinetic parameters: for transitions (yellow arrows) from conducive into active states, λ, and transitions (blue arrows) from active into conducive states, μ (Figure 8A). Consistent with burst frequency control, regulation occurs at two levels: transitioning from inconducive configurations (white) to conducive configurations (light gray), and hence by modulation of 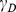 and 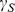, and transitioning from conducive to active promoter states (dark gray), i.e., modulation of λ (Figure 8A). The model allows for nucleosome assembly transitions from active into inconducive promoter states (white) in accordance with our finding that nucleosome formation does not compete with transcription factor binding. All kinetic parameters, including λ and μ, were definitely determined by our data (Figure 8A; Text S1).

As claimed—without further adjustment of parameter values or introduction of new parameters—the model successfully integrated EM and noise data: predictions of configurational probabilities based on the topologies of Figure 4D and of Figure 8A were identical (not shown); and for virtually any smooth path—a straight line for instance—that connects the two parameter vectors 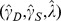 and 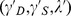 for the *PHO4* wild type and *pho4*Δ mutant, respectively, a noise profile was obtained that naturally fit the observed profile of *PHO5* expression (Figure 8B). In contrast, paths that assumed regulation also of burst size yielded predictions that were inconsistent with experimental observations (Figure 8B).

## Discussion

The chromatin structure of eukaryotic promoters is subject to poorly understood “remodeling” upon transcriptional activation. To clarify the relationship between the structural dynamics of promoter chromatin and gene expression, we here proposed a stochastic model for the *PHO5* gene of yeast (Figure 8A). This model has the following essential properties: (*A*_R_) Assumption of a random process: At steady state, the promoter stochastically transitions between alternative nucleosome configurations; allowed transitions include nucleosome assembly and disassembly transitions between configurations that differ by one nucleosome, and sliding transitions that move nucleosomes out of, but not into, the central N-2 position of the promoter. (*A*_S_) Assumption of a simple process: Nucleosome transition probabilities per time and molecule depend only on the kind of transition—assembly, disassembly and sliding—rather than nucleosome position or configuration. (*A*_C_) Conduciveness hypothesis: Removal of nucleosome N-2 is necessary for transcription. This specifies the nucleosome configurations that are conducive to transcription. (*A*_D_) Assumption of nucleosomal dominance: Nucleosomes exclude transcription factors from promoter DNA [6], but not vice versa. (*A*_F_) Regulatory assumption: The transcriptional activator of *PHO5*, Pho4, stimulates promoter nucleosome disassembly and sliding and, following nucleosome removal, assembly of a scaffold complex of general transcription factors that supports transcription [28].

Together, *A*_R_, *A*_C_, and *A*_D_ imply that transcription occurs in the form of stochastic “bursts” (Figure 1A); the fundamental bursting frequency is determined by the dynamics of promoter nucleosome fluctuations. The essential effect of “chromatin remodeling” is a shift of probability mass from promoter configurations with more to those with fewer nucleosomes. Loss of promoter nucleosomes by accelerated removal (*A*_F_), rather than steric exclusion (*A*_D_), means that nucleosomes are an integral part of the regulatory mechanism for transcription, and not passive repressors whose inhibitory effect is overcome by mass action. However, activated transcription is not explained by accelerated nucleosome removal alone (*A*_F_).

This model has withstood our critical testing, while plausible alternatives could be refuted. Importantly, our findings provide direct structural support for the hypothesis of transcriptional bursting, a concept that has proved useful to account for and mechanistically interpret measurements of gene expression noise [3],[4],[15],[29]–[32]. Other, previous findings may also be explained on our theory, as detailed below.

A critical test of the random process assumption *A*_R_ required the establishment of methods that allowed us to investigate the nucleosome configuration of single *PHO5* gene molecules. Previous analyses of *in vivo* chromatin remodeling relied on endonucleolytic cleavage and averaging over large numbers of gene molecules; both averaging and DNA cleavage erased the information necessary to provide a test of our hypothesis: the probability distribution of promoter nucleosome configurations.

Remarkably, a simple stochastic process in accordance with assumptions *A*_R_ and *A*_S_, and hence two degrees of freedom only, predicted each of the experimentally observed probability distributions with surprising accuracy when based on either the topology of Figure 4D or 4F. Both topologies were overwhelmingly supported by our data against conceivable alternatives (Figure 4G–4I, for instance).

It may be argued that the observed configurational probability distributions of promoter nucleosomes are the product of a deterministic process driven by an extrinsic oscillation, such as the cell cycle, with randomly distributed phase difference between cells, rather than an intrinsically random process, as claimed by *A*_R_. The topology of Figure 4I represents such an alternative process, where transitions between configurations are deterministic, and only the life times of configurations are assumed to be statistically distributed. Our analysis does not exclude the possibility of such an explanation; but it suggests that it would require many more degrees of freedom (kinetic parameters). The model with fewer degrees of freedom is to be preferred, however, not because it might be considered more likely, but because it has greater predictive power and is, therefore, more easily falsifiable.

Some data support the assumption that Pho4 binding stimulates disassembly of only the most proximal nucleosome [22],[33]; accelerated disassembly of nucleosomes in position N-1 would thus require prior removal of nucleosomes in position N-2, which renders UASp2 accessible to activator binding [6]. This assumption may explain the statistical support for the transition topology of Figure 4F against its rival of Figure 4D by some (Figures 4E, 5B), but not all (Figure 5A), datasets for “activated” gene molecules. In contrast, analysis of “repressed” molecules consistently and strongly supported the topology of Figure 4D over 4F (Figure 5C–5E), as expected in the absence of accelerated disassembly. In any case, both topologies provide rather similar statistical predictions for “active” molecules (Figure 4C, 4E), complicating their distinction by statistical means.

The regulatory hypothesis *A*_F_ implies that loss of promoter nucleosomes is a cause of activated transcription, rather than its consequence. Nucleosome disassembly can be strongly dependent on Swi2, the catalytic subunit of the ATP-dependent remodeling enzyme SWI/SNF [34],[35]. Similar observations of genetic dependence, and of activator-remodeler interactions are frequently invoked in support of this assumption. This argument overlooks, however, that the same observations are equally consistent with the opposite assumption—that nucleosome loss is a consequence of promoter activation, possibly due to steric exclusion by transcription factors. Indeed, our data support the notion of continual disassembly of promoter nucleosome even in the absence of activators (Figure 5C–5E); this may explain the rapid binding of newly synthesized histones to transcriptionally “inactive” promoters [36]. A critical test of *A*_F_, therefore, requires the distinction between cause and effect of nucleosome loss. The two-state promoter model (Figure 1A) provided a means for this distinction. Given *A*_R_, *A*_C_, and *A*_D_, *A*_F_ predicts an increase in the frequency of transcriptional bursting, whereas its rival hypothesis implies an increase in burst size. Noise analysis bore out the former expectation (Figure 6), refuting nucleosome loss by mass action.

Additional support for *A*_F_ was provided by our experimental test of *A*_D_ (hypothesis of nucleosomal dominance). Neither mutation of the TATA box, nor absence of the Pho2 transcription factor caused an increase in promoter nucleosome occupancy (compare Figure 4A with 5A, and Figure 5D with 5E, respectively)—again refuting the hypothesis of nucleosome loss by mass action, however corroborating both *A*_D_ and *A*_F_. *A*_D_ may explain the poor correlation between the dwell time of activators at their DNA recognition sequences *in vivo* and their binding affinity [37].

*A*_F_ furthermore implies that activated transcription encompasses accelerated assembly of a scaffold of general transcription factors [28] following nucleosome removal. Alternatively it may be assumed that nucleosome removal is either sufficient for activated transcription [38], or that Pho4 stimulates other steps than scaffold assembly, such as the rate of transcription in the active state, ε. The first of these alternatives is refuted by our finding that nucleosome loss did not quantitatively account for activated transcription. The second alternative implies that *PHO5* transcription is regulated, at least in part, by changes in burst size. For this latter assumption the integrated model of Figure 8A predicted a noise profile that was inconsistent with experimental observations (Figure 8B), refuting this second alternative, too. The same observations, however, corroborated *A*_F_ (Figure 8B).

The discovery of a stalled RNA polymerase at transcriptionally inactive promoters in other eukaryotes [39]–[42] does not contradict the notion that steps prior to the release of stalling are rate-limiting to transcription. The *PHO5* promoter assumes the active state even in the absence of Pho4, releasing bursts of transcripts at low frequency (Figure 7B). This may explain the need for additional mechanisms of regulation, such as stalling, to suppress bursting, which in the case of genetic regulators of embryogenesis [41] are likely to have deleterious consequences. A low probability per time of forming a stalled polymerase-promoter complex under repressing conditions may lead, eventually, to complex formation with near certainty when integrated over a sufficiently long time.

The integrated model of Figure 8A allowed us to infer the time scale of promoter nucleosome transitions from measurements of cell cycle time and mRNA half life, suggesting an average life time for unoccupied promoter nucleosome positions, (1/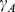), of ∼1 min (Text S2). This estimate is minimally affected by possible error margins for cell cycle time or mRNA half life (Figure S4). An experimental test of this implication of our theory will have to await the development of independent methods for determining the kinetic parameters of nucleosome transitions *in vivo*. We note however that the short half life of unoccupied nucleosome positions may explain the rapid association of newly synthesized histones with promoter DNA, observed on a genome-wide scale [36].

The central role for transcription of the nucleosome in position N-2 (*A*_C_), was imposed by our finding, from noise analysis (Figure 6B), that the total probability of active promoter states is 0.6 under fully activating conditions (*PHO4 pho80*Δ cells), providing a lower bound for the total probability of conducive nucleosome configurations. Our EM data indicated that the only set of configurations consistent with this lower bound, and united by a common structural feature, was the set of configurations with a nucleosome-free position N-2, and not N-1, contrary to previous conjectures [13],[15]. While this conclusion provides a functional explanation for the lower nucleosome occupancy at position N-2 (Figure 4A), it raises the questions of how the requirement for nucleosome removal from position N-2 may be mechanistically explained, and why removal of nucleosomes from position N-1 is not required?

The *PHO5* TATA box resides at the 5′-edge of nucleosomes in position N-1 and, under inducing conditions, is freely accessible in a subset of promoter molecules due to a 3′-directed shift in the position of nucleosomes in this position by about 30 base pairs [14], which was also discernable by EM (analysis not shown). This positional shift and spontaneous partial unwrapping of nucleosomal DNA [7],[43], together with high local concentrations of TBP due to activator-mediated recruitment to the promoter [6], might allow for efficient binding of TBP at the TATA box. Subsequent assembly of the transcription machinery might provide the free energy for further unwrapping of nucleosomal DNA to eventually render the transcription start site accessible, without complete disassembly of the nucleosome [44].

Loss of nucleosomes from position N-2 enables Pho4 binding at UASp2 [6], and may thus render removal or remodeling of the nucleosome in the proximal N-1 position more effective (see above). However, loss of Pho4 binding at UASp2 by mutation of UASp2 did not abolish activated *PHO5* expression; the UASp2 mutant retained ∼25% of its wild type *PHO5* expression (Figure 6B) [45]. In contrast, inhibition of N-2 nucleosome removal by replacement of the N-2 sequence with a strong nucleosome positioning sequence abolished activated transcription entirely [46], as predicted by *A*_C_. Together, these findings point to an inhibitory effect of this nucleosome beyond blocking access to UASp2. A possible explanation is that loss of nucleosome N-2 allows general transcription factors, such as TBP, to slide along the DNA toward the core promoter, following their recruitment by Pho4 to promoter DNA at UASp1. Consistently, bacterial transcription factors find their operator sequence *in vivo* by a combination of three-dimensional diffusion and DNA sliding [47]. This provides a possible explanation for the observation that tethering of a bacterial repressor protein between upstream activation sequences and the TATA box of the *GAL1* promoter of yeast severely inhibits *GAL1* transcription [48]. Nucleosomal inhibition of “promoter scanning” by general transcription factors might also explain the occurrence and position of a “nucleosome free region” at many promoters from yeast to human [49],[50], analogous to the N-2 region of the *PHO5* promoter.

## Materials and Methods

### Strains and Plasmids

Strains and plasmids used in this study are summarized in Table S2. Plasmid pSH17, bearing *TEF2*:*LexA-TAP* and *GAL1*:*R*, was kindly provided by S. Hamperl and J. Griesenbeck.

### Chromatin Ring Purification

Purification of chromatin rings was performed as previously described [14],[17],[51], except that calmodulin affinity purification was performed first, followed by IgG-sepharose affinity purification and TEV cleavage (6His-tagged TEV was a generous gift of V. Thai).

### Trimethylpsoralen Crosslinking and Denaturing

Crosslinking was performed essentially as described [52] with the following modifications. Following chromatin ring elution from the IgG column, samples were pooled and placed onto a 10 cm petri dish that was floating on an ice water slurry, and positioned 5 cm away from five 366 nm UV bulbs in a Stratalinker 2400 (Stratagene); 0.05 volumes of 400 µg/ml trimethylpsoralen was added and the sample was then incubated in the dark on ice for 5 min. Samples were then irradiated by UV for 5 min. Addition of psoralen, incubation in the dark, and crosslinking were performed a total of seven times for each sample. Following crosslinking, the sample was treated with RNaseA for 2 h at 37°C followed by a Proteinase K/SDS treatment for 4 h at 55°C. DNA was extracted with phenol/chloroform and precipitated. DNA was resuspended, digested with NcoI, purified using a DNA Clean and Concentrator kit (ZymoResearch), and eluted from the column with 8 µl of TEN (30 mM TEACl, 20 mM EDTA, 10 mM NaCl). Denaturing, spreading, staining with uranyl acetate, and rotary metal shadowing was performed as previously described [52].

Heterogeneous bubble sizes were due, at least in part, to the sequence specificity of psoralen intercalation, as psoralen preferentially intercalates into dinucleotides TA and AT [53]—resulting either in bubbles that were larger than the expected nucleosome size, when linker DNA failed to crosslink, or in bubbles that were smaller than the expected nucleosome size where no nucleosomes had been present (Figure S1). Because of longer linker lengths, fused nucleosome bubbles occurred seldom on promoter DNA. Smaller than expected bubbles may also have been due to crosslinking of DNA that transiently unspooled from the histone octamer. Bubbles on naked control DNA added to chromatin ring preparations measured 90 base pairs in length, on average. We therefore excluded bubbles smaller than 90 base pairs when counting promoter nucleosome bubbles. (Bubbles attributable to preinitiation complex formation are on the order of ∼60 base pairs [28].) To determine promoter nucleosome positions, we determined the positions of UASp2 (N-2 position), the TATA box, and the transcription start site (N-1 position) for every gene molecule, converting base pair distances into contour length by relating the measured contour length of the entire gene molecule (average over both strands) to the known length of the gene molecule in base pairs. Promoter nucleosome occupancies thus determined were in good agreement with the results of restriction nuclease accessibility assays in nuclei (Figure S2), and isolated chromatin rings [13]. Although we cannot exclude the possibility that smaller than expected bubbles were also due to intermediate structures of nucleosome assembly and disassembly, the paucity of sub-nucleosome size DNA fragments in previous micrococcal nuclease digestions of *PHO5* promoter chromatin suggests that the number of such intermediates is small [14].

### Electron Microscopy

Images were taken on a JEOL 1230 electron microscope at 120 keV at 20,000-fold magnification. Images were processed and analyzed in ImageJ. At least 200 individual *PHO5* molecules were analyzed for each dataset.

### Calculations

Calculations for stochastic gene expression models were based on the following master equation [15]:
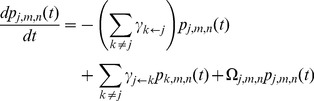
(*ii*)for all *j*, *m*, *n*, where 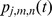 is the probability at time *t* of finding the *PHO5* promoter in state *j*, and the cell with *m* transcript molecules and *n* protein molecules expressed under control of the *PHO5* promoter; and
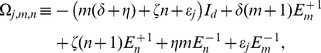
with 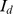 the identity mapping, 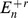 the “step operator” defined by 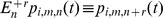, and 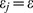 if *j* is an active promoter state, but 0 otherwise. Equation (*i*) may derived from (*ii*) by summing over all *m* and *n* and applying the steady state assumption, 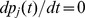 for all *j*. For the two-state model (Figure 1A), equation (*ii*) simplifies to
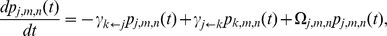
with *k*, *j* = ON, OFF, 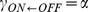, and 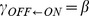. Solutions for steady state noise and mean were obtained analytically, as previously described [15].

### Maximum Likelihood Analysis

Values of the kinetic parameter for nucleosome disassembly, 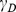, and sliding, 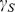, were determined by maximizing the likelihood, *L_T_*, of topology 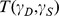 with 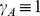, given the EM results *R*:
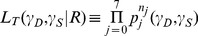
(*iii*)where 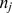 is the number of molecules with promoter nucleosome configuration *j* in *R*, and 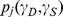 is the theoretical probability of configuration *j* for 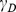 and 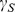 given *T* and the stochastic process model (*ii*). Thus, that parameter hypothesis 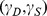 was accepted, which maximizes the probability of the EM data *R* given the stochastic process model (*ii*) and topology *T*.

The statistical *support*, 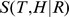, of topology *T* against topology *H* by data *R*, is
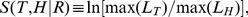
(*iv*)where 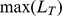 and 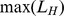 are the maximum likelihoods of topologies *T* and *H* given *R*.

All calculations were performed using Mathematica 8 (Wolfram).

## Supporting Information

Figure S1 **Psoralen crosslinking of naked and nucleosomal PHO5 DNA.** (A) Plasmid pM70.1, which contains the *PHO5* gene ring construct, was cut with restriction enzymes BamHI and NcoI. The 2.25 kb fragment containing the *PHO5* promoter and ORF was purified and used for psoralen crosslinking trials. The orientation of the molecules is unknown due to the absence of LexA adaptor protein and therefore lack of forked 3′-end. (B–F) Examples of single *PHO5* molecules following one to five rounds (B–F, respectively) of psoralen crosslinking. Note the progressive loss of single stranded DNA bubbles as the number of treatments with psoralen increases. Scale bars are 100 nm. (G) Plasmid pM70.1, which contains the *PHO5* gene ring construct, was cut with restriction enzymes BamHI, NcoI, and DraI. These cuts gave two fragments, a 0.65 kb promoter fragment and a 1.6 kb ORF fragment. The orientation of the molecules is unknown (see main text). (H) Naked *PHO5* promoter and ORF fragments were treated with psoralen (×5) and visualized. An example of a full field electron micrograph is shown on the left (scale bar is 500 nm) and two examples each of crosslinked promoter fragment and ORF fragment are shown on the right (scale bars are 100 nm). Black arrowheads point to single stranded DNA bubbles where there was insufficient crosslinking. (I) Linearized, nucleosome-free *PHO5* promoter and ORF fragments were included in each *PHO5* ring preparation as a control for crosslinking efficiency. Shown here is an example of a preparation of linearized, repressed *PHO5* gene rings with added control DNA. Samples were crosslinked after the addition of the control DNA with psoralen (×7) and prepared as described. An example of a full field electron micrograph is shown on the left (scale bar is 500 nm) and two examples of crosslinked promoter fragments and ORF fragments are shown on the right (scale bars are 100 nm). Black arrowheads point to single stranded DNA bubbles that failed to crosslink. White arrowheads point to the forked 3′-ends of naked ORF molecules that were bound by free LexA adaptor proteins in the gene ring preparation. Linearized gene ring molecules are distinguishable from naked *PHO5* promoter and ORF DNA by measuring their relative contour lengths. (J) Single stranded DNA bubble sizes for repressed (light gray) and activated (dark gray) TATA-less *PHO5* gene rings. Bubbles are from the entire *PHO5* molecule including the promoter and ORF regions. The average bubble size for repressed and activated rings was 211 bp and 203 bp, respectively. (K) Single stranded promoter bubble sizes for repressed (light gray) and activated (dark gray) TATA-less *PHO5* gene rings. The average promoter nucleosome bubble size for repressed and activated rings was 153 bp and 115 bp, respectively.
(TIF)

Figure S2 **Endonuclease accessibility and nucleosome occupancies inferred by EM.** Accessibilities of the N-1 and N-2 nucleosome positions were previously measured by restriction enzyme digestion on nuclei preparations in repressed, mutant (*pho4*:Δ85-99), and activated *PHO5* strains (white dots) [15]. Accessibilities for N-1, N-2, and N-3 were measured in our single molecule EM analysis for repressed (light gray bars), mutant (*pho4*:Δ85-99, gray bars), and activated (dark gray bars) *PHO5* gene rings. No restriction sites exist in the N-3 position that were suitable for accessibility assays.
(TIF)

Figure S3
**Excised TATA WT** ***PHO5*** **rings are fully inducible in phosphate-free media.** Cultures of yM2.1 [pSH17] (TATA WT *PHO5* ring strain, *PHO4 PHO80*, containing plasmid pSH17), and yM8.14 [pSH17] (TATA WT *PHO5* ring strain, *PHO4 pho80*Δ, containing plasmid pSH17), were grown in synthetic complete (SC) media made with raffinose as the carbon source and lacking leucine. Phosphatase activity was assayed as previously described [6]. (*PHO5* is constitutively active in yM8.14, due to the *pho80*Δ mutation.) The cultures were split in half. To one half galactose was added (Gal) to a final concentration of 2% to induce the R-recombinase and excision of *PHO5* gene rings. Following 1.5 h of incubation in the presence of galactose, cultures were again assayed for phosphatase activity. Cells were then transferred to phosphate-free SC media containing glucose, rather than galactose, and cultured for another 8 h, during which samples were taken in regular intervals for phosphatase assays. Cells divided approximately once (doubling number 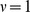) while in phosphate-free media. The final phosphatase activity ratio between yM2.1 and yM2.1+Gal was 1.64, in close agreement with a *PHO5* ring excision efficiency of ∼75% (data not shown) and full *PHO5* induction on the excised rings (the expected ratio for full induction is 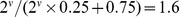). Experiments were performed in triplicate; error bars represent the standard deviations of the measurements.(TIF)

Figure S4
**Error estimation for time scale of promoter nucleosome transitions.** The kinetic parameters for the degradation of protein, ζ, and mRNA, δ, determined by measurement of the average cell cycle time, and mRNA half life, respectively, provide the time scale for promoter nucleosome dynamics. For given values of ζ and δ, the kinetic parameter value for nucleosome assembly, 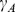, is chosen such that the measured CV^2^ for protein noise in the *PHO4* wild type, 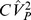, is obtained. If 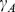 is known, so are 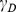 and 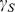, whose values relative to 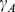 were determined by our EM data (see Table S3). Thus, the value of 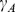 provides a time scale for the kinetics of promoter nucleosome transition. To see how sensitive 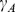 is to variations in the measured values for ζ and δ, 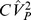 was calculated as a function of mRNA half life (A), or protein half life (B) with all other parameters kept constant. For an error margin of ±5 min for mRNA half life (A), and ±15 min for the protein half life (cell cycle time) (B)—we believe the actual error of measurement is significantly smaller than suggested by these margins—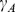 (in transitions min^−1^) was recalculated to again fit the measured value of 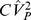 (indicated by the dashed horizontal, yellow line), providing the corresponding variation in 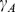 (±).(TIF)

Table S1 **Nucleosome configuration probabilities and nucleosome loss values.** Nucleosome configurations were analyzed in six strains. The number of *PHO5* molecules analyzed for each strain is reported in the “*Rings*” column. The probability of finding *PHO5* gene rings with promoter nucleosome configurations 0 through 7 are shown, as are the observed molecule counts (below probabilities, in brackets). These probabilities, which were determined by counting of nucleosome-size bubbles (Figure S1), were used to calculate the average loss of promoter nucleosomes upon *PHO5* induction relative to full occupancy (Pro. Loss). “*R*-value analysis” was also used to calculate nucleosome loss on the promoter and ORF of activated *PHO5* gene rings relative to repressed gene rings. The *R*-value is defined as the ratio of single stranded DNA and the contour length of the DNA molecule. To quantify the apparent nucleosome loss due to *PHO5* activation (“*R*-Value Loss”), we determined the average extent of single stranded DNA per molecule in base pairs, inferred from knowledge of the total length of the *PHO5* gene ring, promoter, and open reading frame of 2,246, 610, and 1,636 base pairs, respectively, and the EM contour length of the molecule. The *R*-Value Loss was then calculated by taking the (average) difference in single stranded DNA between molecules isolated from *pho4*Δ *pho80*Δ cells (in base pairs) and dividing it by the average bubble size of the repressed promoter (153 bp). The difference of 1.89 nucleosomes between repressed and activated gene rings thus determined closely matched the average linking difference between activated and repressed TATA-less *PHO5* rings of +1.85 [14],[15], indicating that nucleosome disassembly was associated with a linking change of about +1 per nucleosome *in vivo*, in accord with earlier observations for the SV40 chromosome and synthetic chromatin rings [54]. Likewise, the difference of 0.89 nucleosomes between repressed rings and rings isolated from *pho4*:Δ85-99 cells determined by EM was closely similar to the previously determined linking number difference of +0.92 between rings [15].
(PDF)

Table S2 **Strain list.**
(PDF)

Table S3
**Maximum likelihood parameter values.** Relative parameter values for nucleosome disassembly, 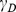, and sliding, 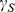, were determined by maximum likelihood analysis of EM data (see Materials and Methods). The superscript “*cyt*” refers to cytoplasmic Pho4, i.e., *PHO4 PHO80* wild type grown in high phosphate. *TATA* and *tata* refer to the wild type and mutant *PHO5 TATA* box, respectively. Parameter values for the integrated model of Figure 8A were 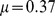 min^−1^ (transition from active to conducive states), 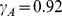 min^−1^ (nucleosome assembly), 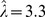 min^−1^ (*PHO4 pho80*Δ *TATA^PHO5^*); and 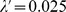 min^−1^ (*pho4*Δ *pho80*Δ *TATA^PHO5^*). The values of other parameters were as indicated for Figure 5B: 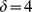 h^−1^, 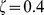 h^−1^, 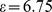 min^−1^, 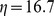 min^−1^. These parameter values were determined, as described in the main text, from RNA-FISH, northern blot analysis, and measurements of protein molecule number and noise. With the nucleosome disassembly and sliding parameters from EM, and 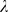 adjusted to account for the observed average transcript number of 7.5 per cell from FISH (unpublished data), the integrated model predicted an intrinsic protein noise value of 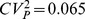, in close agreement with the measured value of 0.068 for the *pho4*[85-99] mutant [15].(PDF)

Text S1 **Definiteness of parameter values for integrated model.**
(DOCX)

Text S2 **Kinetic parameter values.**
(DOCX)

## Abbreviations

CFP: cyan fluorescent protein
*CV^2^*: coefficient of variation
EM: electron microscopy
FISH: fluorescence *in situ* hybridization
TBP: TATA box binding protein
UASp1: upstream activating sequence 1
UASp2: upstream activating sequence 2
UV: ultraviolet
