# Supplementary figures and images for: Linking Stochastic Fluctuations in Chromatin Structure and Gene Expression
Source: PLoS Biol. 2013 Aug 6;11(8):e1001621. doi: 10.1371/journal.pbio.1001621 (PMC3735467; doi:10.1371/journal.pbio.1001621)

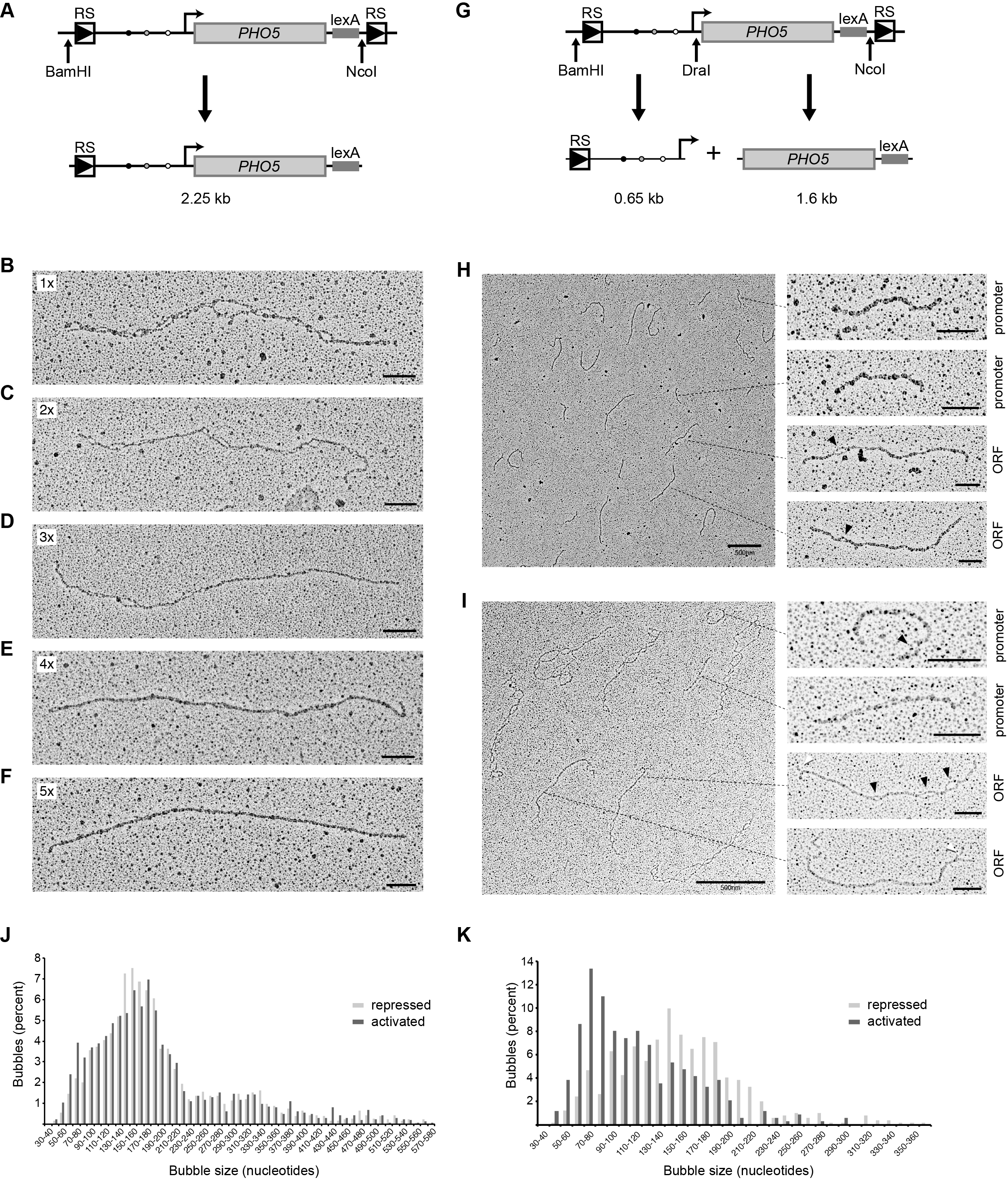

Supplement: Figure S1 — Psoralen crosslinking of naked and nucleosomal PHO5 DNA. (A) Plasmid pM70.1, which contains the PHO5 gene ring construct, was cut with restriction enzymes BamHI and NcoI. The 2.25 kb fragment containing the PHO5 promoter and ORF was purified and used for psoralen crosslinking trials. The orientation of the molecules is unknown due to the absence of LexA adaptor protein and therefore lack of forked 3′-end. (B–F) Examples of single PHO5 molecules following one to five rounds (B–F, respectively) of psoralen crosslinking. Note the progressive loss of single stranded DNA bubbles as the number of treatments with psoralen increases. Scale bars are 100 nm. (G) Plasmid pM70.1, which contains the PHO5 gene ring construct, was cut with restriction enzymes BamHI, NcoI, and DraI. These cuts gave two fragments, a 0.65 kb promoter fragment and a 1.6 kb ORF fragment. The orientation of the molecules is unknown (see main text). (H) Naked PHO5 promoter and ORF fragments were treated with psoralen (×5) and visualized. An example of a full field electron micrograph is shown on the left (scale bar is 500 nm) and two examples each of crosslinked promoter fragment and ORF fragment are shown on the right (scale bars are 100 nm). Black arrowheads point to single stranded DNA bubbles where there was insufficient crosslinking. (I) Linearized, nucleosome-free PHO5 promoter and ORF fragments were included in each PHO5 ring preparation as a control for crosslinking efficiency. Shown here is an example of a preparation of linearized, repressed PHO5 gene rings with added control DNA. Samples were crosslinked after the addition of the control DNA with psoralen (×7) and prepared as described. An example of a full field electron micrograph is shown on the left (scale bar is 500 nm) and two examples of crosslinked promoter fragments and ORF fragments are shown on the right (scale bars are 100 nm). Black arrowheads point to single stranded DNA bubbles that failed to crosslink. White arrowheads point to [file pbio.1001621.s001.tif]

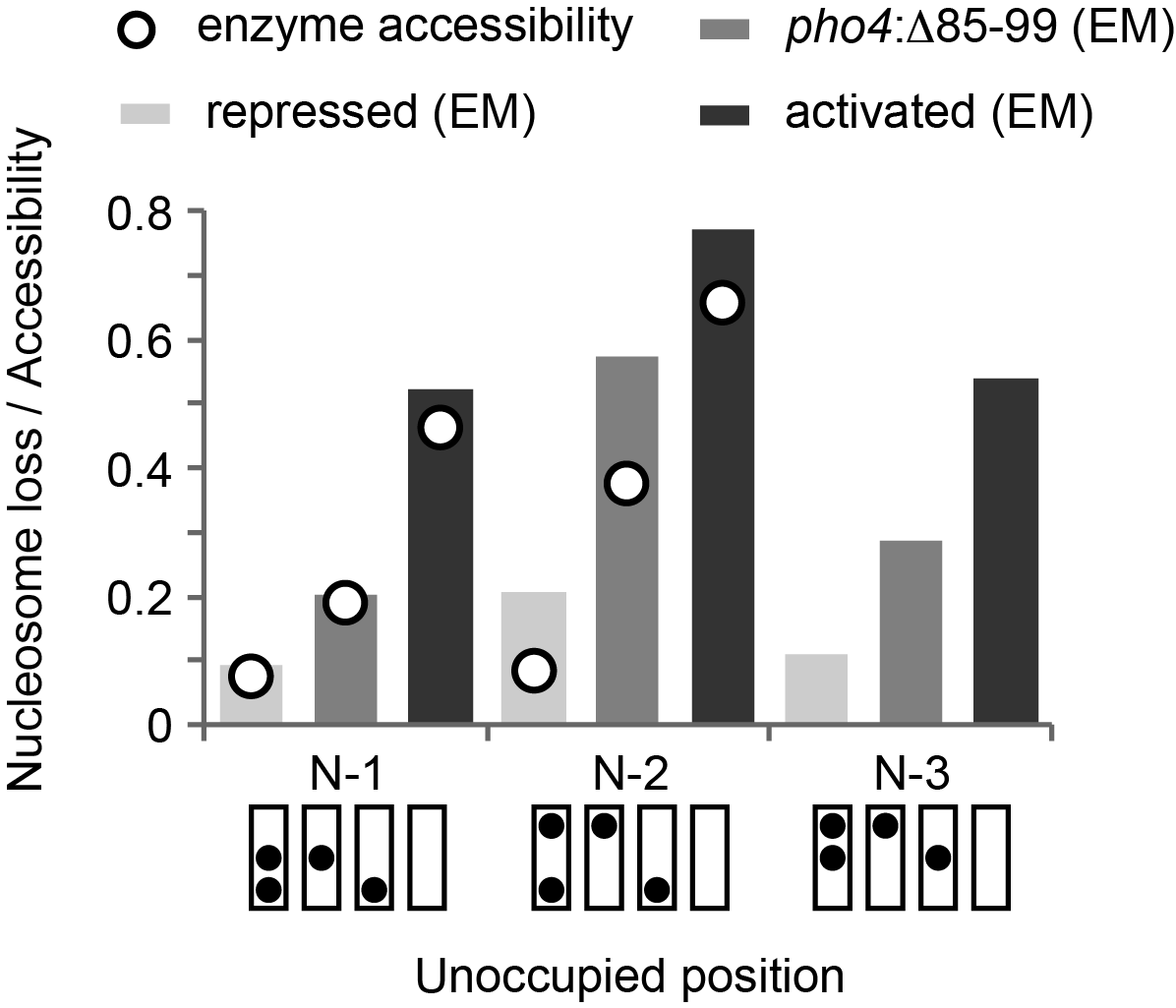

Supplement: Figure S2 — Endonuclease accessibility and nucleosome occupancies inferred by EM. Accessibilities of the N-1 and N-2 nucleosome positions were previously measured by restriction enzyme digestion on nuclei preparations in repressed, mutant (pho4:Δ85-99), and activated PHO5 strains (white dots) [15]. Accessibilities for N-1, N-2, and N-3 were measured in our single molecule EM analysis for repressed (light gray bars), mutant (pho4:Δ85-99, gray bars), and activated (dark gray bars) PHO5 gene rings. No restriction sites exist in the N-3 position that were suitable for accessibility assays. (TIF) [file pbio.1001621.s002.tif]

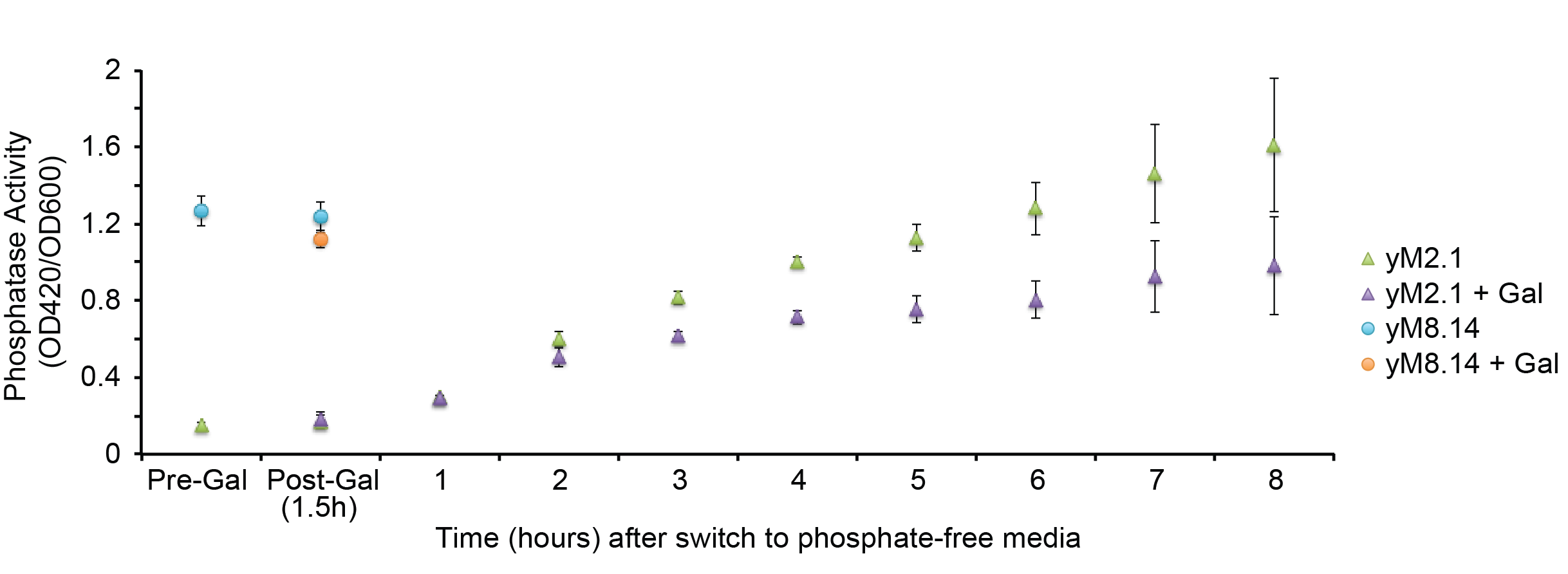

Supplement: Figure S3 — Excised TATA WT PHO5 rings are fully inducible in phosphate-free media. Cultures of yM2.1 [pSH17] (TATA WT PHO5 ring strain, PHO4 PHO80, containing plasmid pSH17), and yM8.14 [pSH17] (TATA WT PHO5 ring strain, PHO4 pho80Δ, containing plasmid pSH17), were grown in synthetic complete (SC) media made with raffinose as the carbon source and lacking leucine. Phosphatase activity was assayed as previously described [6]. (PHO5 is constitutively active in yM8.14, due to the pho80Δ mutation.) The cultures were split in half. To one half galactose was added (Gal) to a final concentration of 2% to induce the R-recombinase and excision of PHO5 gene rings. Following 1.5 h of incubation in the presence of galactose, cultures were again assayed for phosphatase activity. Cells were then transferred to phosphate-free SC media containing glucose, rather than galactose, and cultured for another 8 h, during which samples were taken in regular intervals for phosphatase assays. Cells divided approximately once (doubling number ) while in phosphate-free media. The final phosphatase activity ratio between yM2.1 and yM2.1+Gal was 1.64, in close agreement with a PHO5 ring excision efficiency of ∼75% (data not shown) and full PHO5 induction on the excised rings (the expected ratio for full induction is ). Experiments were performed in triplicate; error bars represent the standard deviations of the measurements. (TIF) [file pbio.1001621.s003.tif]

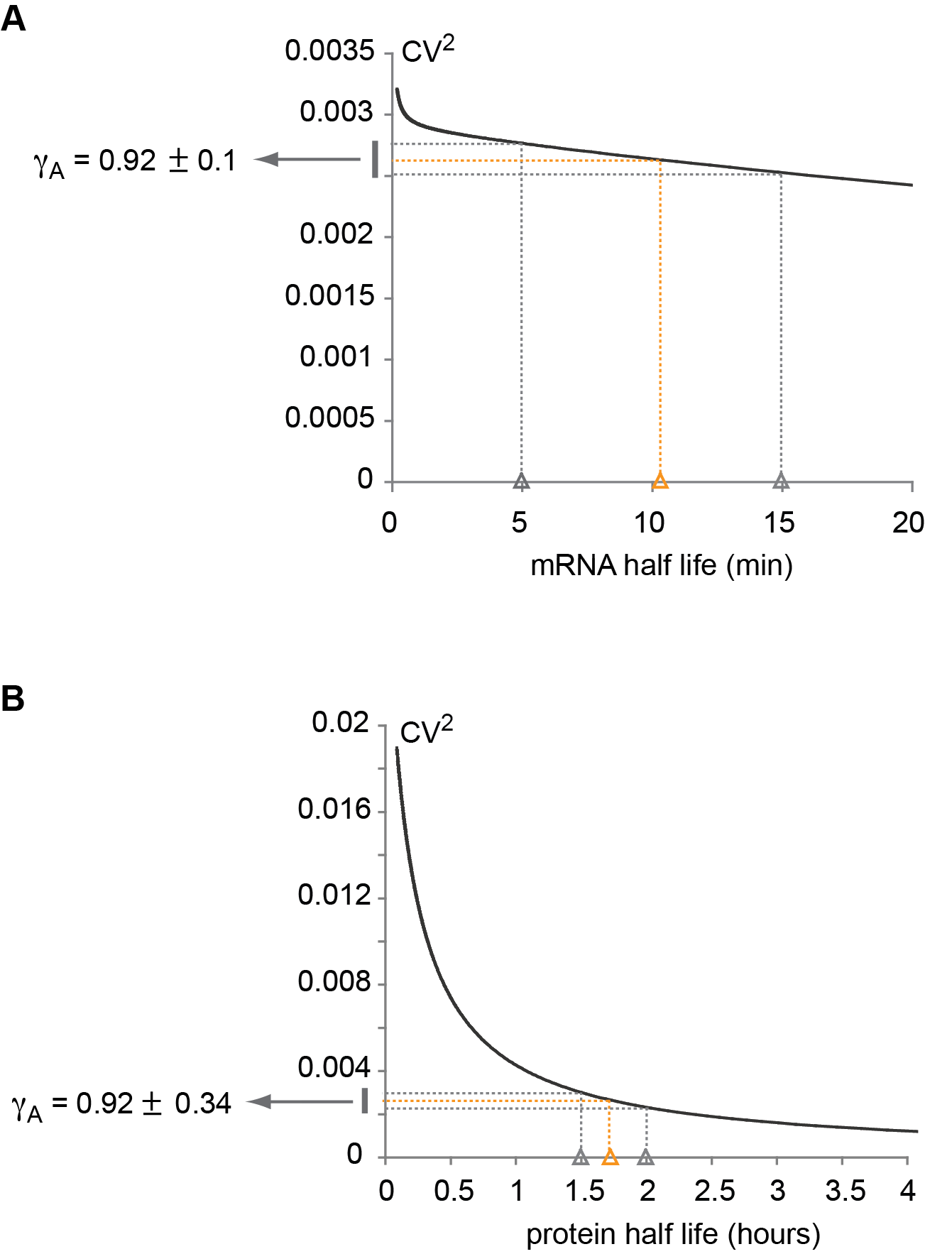

Supplement: Figure S4 — Error estimation for time scale of promoter nucleosome transitions. The kinetic parameters for the degradation of protein, ζ, and mRNA, δ, determined by measurement of the average cell cycle time, and mRNA half life, respectively, provide the time scale for promoter nucleosome dynamics. For given values of ζ and δ, the kinetic parameter value for nucleosome assembly, , is chosen such that the measured CV2 for protein noise in the PHO4 wild type, , is obtained. If is known, so are and , whose values relative to were determined by our EM data (see Table S3). Thus, the value of provides a time scale for the kinetics of promoter nucleosome transition. To see how sensitive is to variations in the measured values for ζ and δ, was calculated as a function of mRNA half life (A), or protein half life (B) with all other parameters kept constant. For an error margin of ±5 min for mRNA half life (A), and ±15 min for the protein half life (cell cycle time) (B)—we believe the actual error of measurement is significantly smaller than suggested by these margins— (in transitions min−1) was recalculated to again fit the measured value of (indicated by the dashed horizontal, yellow line), providing the corresponding variation in (±). (TIF) [file pbio.1001621.s004.tif]
