# Supplementary material for: Linking Stochastic Fluctuations in Chromatin Structure and Gene Expression
Source: PLoS Biol. 2013 Aug 6;11(8):e1001621. doi: 10.1371/journal.pbio.1001621 (PMC3735467; doi:10.1371/journal.pbio.1001621)
Supplement: Table S1 — Nucleosome configuration probabilities and nucleosome loss values. Nucleosome configurations were analyzed in six strains. The number of PHO5 molecules analyzed for each strain is reported in the “Rings” column. The probability of finding PHO5 gene rings with promoter nucleosome configurations 0 through 7 are shown, as are the observed molecule counts (below probabilities, in brackets). These probabilities, which were determined by counting of nucleosome-size bubbles (Figure S1), were used to calculate the average loss of promoter nucleosomes upon PHO5 induction relative to full occupancy (Pro. Loss). “R-value analysis” was also used to calculate nucleosome loss on the promoter and ORF of activated PHO5 gene rings relative to repressed gene rings. The R-value is defined as the ratio of single stranded DNA and the contour length of the DNA molecule. To quantify the apparent nucleosome loss due to PHO5 activation (“R-Value Loss”), we determined the average extent of single stranded DNA per molecule in base pairs, inferred from knowledge of the total length of the PHO5 gene ring, promoter, and open reading frame of 2,246, 610, and 1,636 base pairs, respectively, and the EM contour length of the molecule. The R-Value Loss was then calculated by taking the (average) difference in single stranded DNA between molecules isolated from pho4Δ pho80Δ cells (in base pairs) and dividing it by the average bubble size of the repressed promoter (153 bp). The difference of 1.89 nucleosomes between repressed and activated gene rings thus determined closely matched the average linking difference between activated and repressed TATA-less PHO5 rings of +1.85 [14],[15], indicating that nucleosome disassembly was associated with a linking change of about +1 per nucleosome in vivo, in accord with earlier observations for the SV40 chromosome and synthetic chromatin rings [54]. Likewise, the difference of 0.89 nucleosomes between repressed rings and rings isolated from pho4:Δ85-99 cells deter [file pbio.1001621.s005.pdf]

Strain	*PHO5* State	Relevant Genotype	TATA box	Rings Analyzed	Promoter Nucleosome Configuration Probabilities and [Molecule Counts]								Pro.	R-Value			R-Value Nuc. Loss		
					0	1	2	3	4	5	6	7	Loss	Total	Pro.	ORF	Total	Pro.	ORF
**yM63.19**	repressed	*pho4Δ, pho80Δ*	mutant	210	0.671 [141]	0.048 [10]	0.138 [29]	0.071 [15]	0.029 [6]	0.005 [1]	0.033 [7]	0.005 [1]	0.41	0.863	0.741	0.908	-	-	-
**yM89.1**	partially activated	*pho4[85-99], pho80Δ*	mutant	203	0.3 [61]	0.039 [8]	0.281 [57]	0.079 [16]	0.138 [28]	0.01 [2]	0.094 [19]	0.059 [12]	1.06	0.802	0.6	0.877	0.89	0.56	0.33
**yM19.2**	activated	*PHO4, pho80Δ*	mutant	213	0.066 [14]	0.038 [8]	0.183 [39]	0.052 [11]	0.178 [38]	0.075 [16]	0.174 [37]	0.235 [50]	1.83	0.733	0.374	0.867	1.89	1.46	0.43
**yM2.1**	repressed	*PHO4, PHO80*	wild-type	210	0.595 [125]	0.119 [25]	0.133 [28]	0.043 [9]	0.033 [7]	0.01 [2]	0.043 [9]	0.024 [5]	0.54	0.841	0.702	0.893	-	-	-
**yM8.14**	activated	*PHO4, pho80Δ*	wild-type	203	0.074 [15]	0.02 [4]	0.177 [36]	0.064 [13]	0.212 [43]	0.025 [5]	0.182 [37]	0.246 [50]	1.84	0.749	0.383	0.885	1.37	1.28	0.09
**yM156.1**	repressed	*PHO4, PHO80, pho2Δ*	wild-type	209	0.522 [109]	0.096 [20]	0.196 [41]	0.057 [12]	0.062 [13]	0.024 [5]	0.033 [7]	0.01 [2]	0.617	0.836	0.693	0.889	-	-	-
