# Supplementary material for: Linking Stochastic Fluctuations in Chromatin Structure and Gene Expression
Source: PLoS Biol. 2013 Aug 6;11(8):e1001621. doi: 10.1371/journal.pbio.1001621 (PMC3735467; doi:10.1371/journal.pbio.1001621)
Supplement: Table S2 — Strain list. (PDF) [file pbio.1001621.s006.pdf]

Strain	Parental Strain	Plasmid	Relevant Genotype	Source
yM2.1	yM1.12	pM53.1	*PHO4, PHO80, PHO5[GC, TATA]*	Boeger et al., 2003
yM8.14	yM2.1	pM67.6	*PHO4, pho80::HIS3, PHO5[GC, TATA]*	Boeger et al., 2003
yM19.2	yM17.3	pM70.1	*PHO4, pho80::HIS3, PHO5[GC, tata]*	Boeger et al., 2003
yM63.19	yM19.2	pCM4.5	*pho4::URA3, pho80::HIS3, PHO5[GC, tata]*	Mao et al., 2010
yM89.1	yM63.19	pCM61.5	*pho4:Δ85-99, pho80::HIS3, PHO5[GC, tata]*	Mao et al., 2010
yM156.1	yM2.1	pCM118.1	*PHO4, PHO80, PHO5[GC, TATA], pho2::KanMX*	This study
yE2.1	EY2343	pCM43.12	*PHO4, pho80::LEU2, PHO5p:CFP-KanR*	Mao et al., 2010
yE4.1	yE2.1	pCM4.5	*pho4::URA3, pho80::LEU2, PHO5p:CFP-KanR*	Mao et al., 2010
		pSH17	*TEF2p:LexA-TAP, GAL1p:R-Recombinase (Z. rouxii), LEU2-RS*	S. Hamperl (U. Regensberg)
