# Supplementary material for: Linking Stochastic Fluctuations in Chromatin Structure and Gene Expression
Source: PLoS Biol. 2013 Aug 6;11(8):e1001621. doi: 10.1371/journal.pbio.1001621 (PMC3735467; doi:10.1371/journal.pbio.1001621)
Supplement: Table S3 — Maximum likelihood parameter values. Relative parameter values for nucleosome disassembly, , and sliding, , were determined by maximum likelihood analysis of EM data (see Materials and Methods). The superscript “cyt” refers to cytoplasmic Pho4, i.e., PHO4 PHO80 wild type grown in high phosphate. TATA and tata refer to the wild type and mutant PHO5 TATA box, respectively. Parameter values for the integrated model of Figure 8A were min−1 (transition from active to conducive states), min−1 (nucleosome assembly), min−1 (PHO4 pho80Δ TATAPHO5); and min−1 (pho4Δ pho80Δ TATAPHO5). The values of other parameters were as indicated for Figure 5B: h−1, h−1, min−1, min−1. These parameter values were determined, as described in the main text, from RNA-FISH, northern blot analysis, and measurements of protein molecule number and noise. With the nucleosome disassembly and sliding parameters from EM, and adjusted to account for the observed average transcript number of 7.5 per cell from FISH (unpublished data), the integrated model predicted an intrinsic protein noise value of , in close agreement with the measured value of 0.068 for the pho4[85-99] mutant [15]. (PDF) [file pbio.1001621.s007.pdf]

**Figure**	**4E**	**5A**	**5B**	**5C**	**5D**	**5E**
**Genotype**	*PHO4 pho80 TATA*	*PHO4 pho80 tata*	*Pho4[85-99] pho80 tata*	*pho4 pho80 tata*	$PHO4^{cyt}$ *pho2 TATA*	$PHO4^{cyt}$ *PHO80 TATA*
**Topology**	Fig. 4F	Fig. 4D	Fig. 4F	Fig. 4D	Fig. 4D	Fig. 4D
γ_D / γ_A	1.8	1.56	0.68	0.15	0.25	0.23
γ_S / γ_A	3.64	1.7	2.04	0.64	0.45	0.36
