# Supplementary material for: Linking Stochastic Fluctuations in Chromatin Structure and Gene Expression
Source: PLoS Biol. 2013 Aug 6;11(8):e1001621. doi: 10.1371/journal.pbio.1001621 (PMC3735467; doi:10.1371/journal.pbio.1001621)
Supplement: Text S1 — Definiteness of parameter values for integrated model. (DOCX) [file pbio.1001621.s008.docx]

**Supporting Text S1.** *Definiteness of Parameter Values for Integrated Model*

The total probability of promoter states lacking a nucleosome at position N-2, $\hat{p}_{C}$, is provided by our EM data, and $\hat{p}_{ON}$ is determined by the steady state noise profile (Fig. 5B). Hence, $\hat{p}_{ON\left|C\right.}$ is known too, for $p_{ON}=p_{ON\left|C\right.}p_{C}$. Thus, the values of $\hat{p}_{ON}$, $\hat{p}_{ON\left|C\right.}$, and $C\hat{V}_{p}^{2}$ are all known from measurements and all three may be considered functions of *λ*, *μ*, and $\gamma_{A}$. (If $\gamma_{A}$ is known, so are $\gamma_{D}$ and $\gamma_{S}$, whose values relative to $\gamma_{A}$ are determined by our EM data.) Thus, three equations for three unknowns, *λ*, *μ*, and $\gamma_{A}$, are provided. For instance, at steady state, the rates of transitions into and out of active states are equal, i.e. $\lambda(1-p_{ON})p_{C}=(\mu+\gamma_{A})p_{ON\left|C\right.}p_{C}$. Hence, $p_{ON\left|C\right.}=\frac{\lambda}{\lambda+\mu+\gamma_{A}}$.
