# Supplementary material for: Linking Stochastic Fluctuations in Chromatin Structure and Gene Expression
Source: PLoS Biol. 2013 Aug 6;11(8):e1001621. doi: 10.1371/journal.pbio.1001621 (PMC3735467; doi:10.1371/journal.pbio.1001621)
Supplement: Text S2 — Kinetic parameter values. (DOCX) [file pbio.1001621.s009.docx]

**Supporting Text S2.** *Kinetic Parameter Values*

Parameter values for the integrated model of Fig. 8A were $\mu=0.37$ min^-1^ (transition from active to conducive states), $\gamma_{A}=0.92$ min^-1^ (nucleosome assembly), $\hat{\gamma}_{D}=1.45$ min^-1^ (nucleosome disassembly), $\hat{\gamma}_{S}=3.5$ (nucleosome sliding), $\hat{\lambda}=3.3$ min^-1^ (the hat ^ refers to molecules isolated from *PHO4* *pho80*Δ *TATA^PHO5^* cells); and $\gamma'_{D}=0.14$ min^-1^, $\gamma'_{S}=0.58$ min^-1^, $\lambda'=0.025$ min^-1^ (the prime refers to *pho4*Δ *pho80*Δ *tata^PHO5^* cells). The values of other parameters were as indicated for Fig. 5B: $\delta=4$ h^-1^, $\zeta=0.4$ h^-1^, $\varepsilon=6.75$ min^-1^, $\eta=16.7$ min^-1^. The parameter values were determined, as described in the main text, from EM, RNA-FISH, northern blot analysis, and measurements of protein molecule number; with $\lambda'$ as indicated above, which gives $\langle R\rangle=0.4$ (Fig. 6B).
